# The Role of Optical Imaging in Translational Nanomedicine

**DOI:** 10.3390/jfb13030137

**Published:** 2022-08-31

**Authors:** Evelien Hesemans, Kiana Buttiens, Bella B. Manshian, Stefaan J. Soenen

**Affiliations:** 1 NanoHealth and Optical Imaging Group, Translational Cell and Tissue Research Unit, Department of Imaging and Pathology, KU Leuven, Herestraat 49, B3000 Leuven, Belgium; 2 Translational Cell and Tissue Research Unit, Department of Imaging and Pathology, KU Leuven, Herestraat 49, B3000 Leuven, Belgium; 3 Leuven Cancer Institute, 3000 Leuven, Belgium

**Keywords:** optical imaging, bioluminescence, fluorescence, nanomedicines, nanoparticles, biodistribution

## Abstract

Nanomedicines have been a major research focus in the past two decades and are increasingly emerging in a broad range of clinical applications. However, a proper understanding of their biodistribution is required to further progress the field of nanomedicine. For this, imaging methods to monitor the delivery and therapeutic efficacy of nanoparticles are urgently needed. At present, optical imaging is the most common method used to study the biodistribution of nanomaterials, where the unique properties of nanomaterials and advances in optical imaging can jointly result in novel methods for optimal monitoring of nanomaterials in preclinical animal models. This review article aims to give an introduction to nanomedicines and their translational impact to highlight the potential of optical imaging to study the biodistribution of nanoparticles and to monitor the delivery and therapeutic efficacy at the preclinical level. After introducing both domains, the review focuses on different techniques that can be used to overcome some intrinsic limitations of optical imaging and how this can specifically benefit nanoparticle studies. Finally, we point out some important key features of nanoparticles that currently hinder their full potential in the clinic and how the advances in optical imaging can help to provide us with the information needed to further boost the clinical translation and expand the field of nanomedicines.

## 1. Introduction

In the last decades, nanomedicines (NMs) have clearly conquered an important place in various biomedical applications across different fields. This wide interest is presumably a direct consequence of their unique physicochemical properties compared to conventionally used formulations. However, the clinical translation of NMs is hampered by the multiple challenges NMs face upon entering the market. Examples of some important key points that need to be addressed to accelerate the clinical translation include, among others, the commercial and practical feasibility, the clinical development feasibility, the translation of preclinical efficacy to clinical outcome, the bridging of preclinical toxicology to patient safety and lastly the proper management of chemistry, manufacturing and quality control [1]. In view of the commercial and practical feasibility, one should consider the potential for improved patient benefit as well as the size of the ultimate target population which will both eventually determine whether the high price needed to make a nanodrug commercially viable is ever going to be paid for. The second challenge, i.e., the clinical development feasibility, refers to a proper clinical study design where large trial populations and long studies are often required to prove superiority over the standard of care, also including the more clinically relevant outcomes such as improved disease modulation, better long-term remission, longer delay of disease progression and better safety with equal efficacy. In addition, the translation of preclinical efficacy to clinical outcome seems to be more complicated for nanomedicines specifically compared with other therapeutics due to greater response rate variability and difficulties in therapeutic efficacy prediction. Nanomedicines tend to be more prone to a lack of predictability of patient benefit which may be the result of the importance of in vivo performance determinants such as pharmacokinetics, tissue distribution, target site accumulation, and penetration which are very different in animal models. In this regard, optical imaging can contribute to solving these challenges where it can be applied to study, among other aspects, the biodistribution, characterization, therapeutic efficacy, and delivery of nanoparticle (NP) formulations. First, we will give a short introduction to nanoparticles (NPs) as promising formulations to revolutionize the health care approach. Here, it will become clear why they received so much attention in research during the last decades. We will highlight their current application potential in the clinic and point out their presence in ongoing clinical trials. In the next section, we will briefly explain the characteristics of optical imaging and we will show the interesting possibilities that this imaging technique has to offer in promoting the clinical translation of NMs. Finally, additional improvements and future perspectives are described to further advance the widespread potential of NMs in shaping and improving the healthcare approach.

## 2. Nanoparticles in Biomedical Applications and Current Limitations

In today’s health care, NPs are used in various biomedical applications ranging from targeted drug delivery, thermal therapy including photoablation, and magnetic hyperthermia to bioimaging and biosensors [2,3]. From the late nineties on, these types of formulations have acquired a unique place in drug delivery and as imaging systems, mainly explained by some significant advantages compared to their free drug counterparts. First of all, NPs can function as a shell around drugs protecting them in biological environments. Encapsulation of therapeutics in NPs provides an additional barrier against environmental factors, as for example, enzyme or pH-dependent degradation [4]. Furthermore, encapsulation inside NPs allows the administration of drugs that would be too toxic in their free drug form and also offers the possibility to develop controlled-release formulations. [4,5]. Another reason why NPs are increasingly being used is their small size, generally <100 nm. It has been shown that the NP size is one of the most important parameters influencing biodistribution, blood circulation half-life, targeting ability, tumor penetration, and the cellular uptake [6]. The nanosize range of NPs makes them almost ideally suited for increasing blood circulation times compared to other agents. Smaller agents, such as free drugs, can often diffuse across cell membranes and spread throughout tissues or can be cleared by the kidneys in relatively short time frames. Larger particles (micron-sized) are rapidly opsonized and are unable to pass through the smallest veins, resulting in rapid clearance of these large particles from the blood. NPs are therefore well suited to prolong blood circulation times of drugs by slowing down particle clearance from the blood, while also impeding drug diffusion throughout different tissues.

For delivery of NPs to neoplasm, NPs are passively targeted towards the tumor via the enhanced permeation and retention (EPR) effect resulting from the small NP size in combination with an increased tumor vascular permeability and poor lymphatic drainage associated with the tumor microenvironment. The EPR effect leads to preferential accumulation of NPs into tumor tissue making these NP formulations specifically interesting for cancer therapy. The small size enables them to leave the blood circulation through leaky blood vessels with tumors, enter the tumor mass itself and achieve a higher intratumoral drug concentration compared to drug concentrations in normal tissues [7,8]. The EPR effect exerted by these formulations reduces the toxicity of the associated chemotherapeutics and is probably, alongside other advantages, part of the reason for the improved efficacy in comparison with the free drug form [4]. In addition, their large surface area to volume ratio, arising from their small size, can easily be modified with functional groups to alter their biochemical properties [5].

While NPs used as molecular imaging agents benefit from rapid clearance of remaining particles in the circulation to reduce the background signal, longer circulation times are preferred for NPs designed as delivery vehicles [9]. One important strategy that is commonly used to prolong the blood circulation time is grafting polyethylene glycol (PEG) onto the NP surface. This hydrophilic polymer forms a hydration shell acting as a steric stabilizer and thereby reducing nonspecific protein–protein and protein–cell interactions and the uptake by the mononuclear phagocyte system (MPS). Apart from increasing the circulation time, it has been shown that PEGylation can also positively influence safety and efficacy, and can furthermore be exploited to reduce the immunogenicity of diverse formulations [10].

In contrast to the passive targeting strategy, guided by the EPR effect, NPs can also be modified with various ligands to actively target the tissue of interest resulting in an increased specific delivery. Antibodies, antibody fragments, aptamers, and peptides are most often used for this active targeting strategy [6]. Additionally, stimuli-responsive functions, stemming from NPs characterized by surface plasmon resonance or magnetic properties, can also be used for targeting [4].

NPs can be categorized based on different parameters including: (1) their function: where they can serve as carrier for therapeutic compounds or used for their own direct toxic effects, (2) their composition: either organic (polymeric, liposomes, micelles, etc.), or inorganic (gold, silica, iron oxide, etc.), or (3) their way of degradation: where they are either biodegradable or non-biodegradable. Biodegradable NPs often consist of polymers releasing the attached or encapsulated compound after enzymatic hydrolysis in a biological environment. This type of NP is commonly chosen for their possibility to control drug release kinetics, their versatility in material production processes and their high drug loading capacity. Polysaccharides and proteins such as dextran, albumin, and collagen are naturally degradable polymers that have already been used as carriers in delivery systems. Non-biodegradable NPs are mostly either silica or metallic based and may, due to their low degradability, result in low clearance rates that can lead to long persistent presence in the body, and associated potential dangers [11]. Yet, this class of NPs can offer quite a few other advantages over biodegradable NPs, mainly owing to intrinsic properties of their composition. For example, metallic NPs can often be well suited for photothermal therapy or serve as contrast agents for bioimaging. In the case of silica NPs, porosity, and differences in pore size and hydrophilicity/hydrophobicity can be easily controlled during manufacturing and allows for excellent loading capacities as well as allowing oxygen diffusion in both directions while at the same time preventing leakage of the entrapped compound [5].

**Figure 1:**
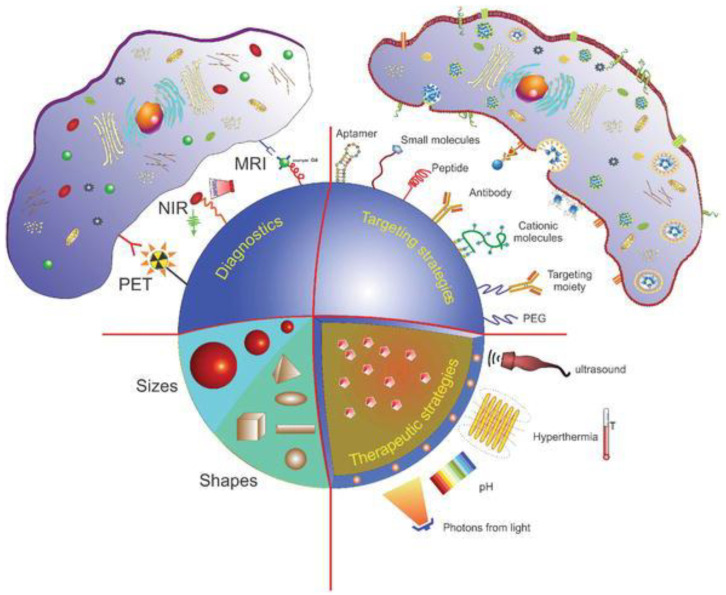
Schematic representation of different strategies to design multifunctional nanoparticles. The structure of a nanocarrier allows the incorporation of one or multiple therapeutic molecules. These NPs can be found in different sizes and shapes. NPs can be actively targeted via the attachment of targeted-specific ligands such as antibodies, antibody fragments, aptamers, and peptides at their surface. Depending on the kind of application, various compounds can be added to turn the nanocarrier into a responsive device to a specific stimulus such as temperature, pH, or magnetic and ultrasound fields. Imaging or contrast agents such as magnetic resonance imaging (MRI), near-infrared (NIR), and/or polyethylene terephthalate (PET) compounds can also be incorporated into a single platform to enable imaging and releasing of drugs from NPs. This figure has been reproduced with permission from Vieira et al. [12] © 2018, IntechOpen.

Through the modification of different parameters, one can obtain multifunctional NPs that can be used for various purposes at the same time including targeting, imaging, triggering the release of therapeutic drugs, and eliminating tumor cells (Figure 1) [8]. These specific characteristics contribute to their broad application potential and can be exploited to improve the efficiency of various types of therapies [5]. Given the wide range of NPs, their possible modifications and the respective impact thereof on their biodistribution and blood circulation times, developing methods to optimize NP delivery efficacy has become a topic of high scientific interest.

### 2.1. Clinical Use

Over the past years, the US Food and Drug Administration (FDA) and European Medicines Agency (EMA) have already approved diverse administration routes for the delivery of NPs including oral, local, topical, and systemic administration. Due to the positive results of numerous clinically approved NP formulations, a lot of preclinical and clinical research focuses on further improving the targeted delivery and efficacy of mainly intravenously administered NPs [4].

From 2013 to 2016, the number of clinical trials focusing on NPs, as well as the number of FDA-approved NMs, has increased [9]. Moreover, since 2016, three new NP formulations, VYXEOS, Patisiran, and Hensify received approval, and eighteen new NP formulations, mainly using liposomes, are investigated in clinical trials. Remarkably, regarding the latter, all except one focus on cancer where fifteen are indicated for treatment and two for imaging [13]. These figures clearly demonstrate that the interest in the field of NMs continues to bloom. Clinical applications of NPs cover a wide variety of disciplines including drug delivery, imaging and diagnostics, phototherapy, vaccine development, and antimicrobial therapy. Here, the NPs are mostly used as delivery vehicles for other therapeutic compounds or as mediators for external stimuli.

#### 2.1.1. Nanoparticles as Delivery Vehicles

##### Cancer Therapy

Many promising therapeutic compounds suffer from low availability and suboptimal biodistribution. In this regard, NPs can serve as carriers to improve their pharmacodynamic and kinetic properties where the compounds can either be entrapped in the core, embedded in the shell, or attached to the surface [5].

In 1995, the FDA approved the use of Doxil as the first cancer NM, composed of doxorubicin, a chemotherapeutic, covered in a protective coating of PEGylated lipids [10]. One can definitely state that Doxil has conquered the status of the gold standard in the field of NMs [10]. Soon after its adoption for clinical use, liposomal formulations of other chemotherapeutics such as DaunoXome [14], Marqibo [15], and Myocet [16] found their way into clinical practice [4].

##### Imaging and Diagnostics

Several NP formulations are also clinically approved for their application in imaging and diagnostics. Early detection and diagnosis of pathological conditions are of utmost importance, particularly for cancer where it was shown that the two-year survival rate for gastrointestinal cancer patients and the ten-year mortality rate for breast cancer patients were positively influenced by early detection. Various imaging modalities such as computed tomography (CT), magnetic resonance imaging (MRI), and positron emission tomography (PET) are employed to detect diseases as early as possible. Unfortunately, conventional contrast agents are often small molecules with a non-specific distribution that are quickly metabolized and sometimes even exert potentially toxic effects. To overcome these drawbacks, NPs are widely explored as imaging contrast agents to support early diagnosis [6]. While NPs showing innate magnetic responsiveness, such as iron oxide NPs, can be effectively used as contrast agents for MRI, NPs taking the form of micron-sized microbubbles can serve as ultrasound-enhancing agents. Until today, only a few NP formulations have been approved as ultrasound contrast agents including Definity and SonoVue, consisting of fluorocarbons and sulfur hexafluoride encapsulated by lipid shells, respectively [4].

##### Other Applications

Apart from applications in cancer, imaging, and diagnostics, NPs are also clinically approved as iron replacement therapies, anesthetics, and fungal and parasitic treatments. Iron oxide colloids increase the iron concentration in the body and are used as a treatment for iron deficient anemia. Currently approved drugs for this application include CosmoFer [4], given intravenously or by intramuscular injection [17], and Venofer [4], administered intravenously [18]. The first general anesthetic NP formulation approved by the FDA is Diprivan, a liposomal-based formulation of Propofol. AmBisome was designed and approved by the FDA for the treatment of fungal infections. This formulation contains Amphotericin B encapsulated in liposomes to reduce the toxicity [4]. One example that even further expands the application area of NPs is Estrasorb, the micellar formulation of estradiol and furthermore the only FDA-approved micelle, used as a topical treatment for moderate to severe vasomotor symptoms of the menopause [9].

Given that PEGylation is a common strategy to influence the blood circulation time of a therapeutic compound, as mentioned before, many FDA-approved NMs are modified with PEG including PEGylated granulocyte colony-stimulating factor (Neulasta) for the treatment of chemotherapy-induced neutropenia, PEGylated anti-hemophilic factor VIII (Adynovate) for treatment of hemophilia A and PEGylated interferon gamma beta-1a (Plegridy) for treatment of relapsing multiple sclerosis [9]. While polymers are often used to increase blood circulation time, they also offer the possibility to design controlled-release formulations. Leuprolide, a testosterone inhibitor, incorporated into slowly degradable polylactide-co-glycolic acid (PLGA) NPs, is approved under the name Eligard as symptomatic treatment of prostate cancer [9].

##### Photothermal and Photodynamic Therapy

Currently, more than 1000 photosensitizers are known for photothermal and photodynamic therapy, derived either from natural or synthetic origin. Many, however, suffer from poor water solubility and limited light-penetration depth. In this regard, NPs can serve as carriers to deliver photosensitizers to tumor tissue via the EPR [19]. In this phototherapeutic approach, local heat and free radicals are produced upon electromagnetic irradiation of the photosensitizer in photothermal and photodynamic therapy, respectively. Local hyperthermia can be induced by various types of electromagnetic radiation, including laser light and microwaves, and causes swelling of mitochondria, protein denaturation, rupture of the cell membrane, and other unfavorable effects. These events can eventually lead to cell death and subsequent uptake by macrophages [20]. On the other hand, photodynamic therapy also involves tissue oxygen in addition to a photosensitizer and a light source. Upon irradiation, cytotoxic reactive oxygen species (ROS) are produced, such as singlet molecular oxygen, hydroxyl radicals, and superoxide anions. Abundant ROS leads to oxidative stress, and damage of cellular macromolecules including lipids, proteins, and nucleic acids and may result in cell death via apoptosis, necrosis, and/or autophagy. Whether these mechanisms are activated separately or simultaneously and which mechanism eventually leads to cell death and tumor destruction depends to some extent on the subcellular localization of the photosensitizer [21]. Apart from the direct phototoxic effect, photodynamic therapy (PDT) can also indirectly eradicate tumor cells by targeting the tumor vasculature, causing oxygen deficiency due to insufficient blood supply, or activating the immune system to attack the cancer cells [5]. The application of PDT is by no means limited to the treatment of cancer since photosensitizers can also accumulate in other non-cancerous pathologic tissue. A wide variety of diseases including hyperplasia, metaplasia, and inflammation can be treated as well with this approach. In addition, due to its antibacterial effect, PDT can also be used as a treatment for a variety of other diseases including acne, psoriasis, and herpes [19]. To give an example, Visudyne, consisting of verteporfin encapsulated in liposomes, is approved for the treatment of age-related macular degeneration where it results in local damage of the endothelium and blockage of the blood vessels upon light stimulation [4]. As verteporfin has a tendency to undergo self-aggregation in aqueous media, loading within liposomes is essential to ensure an adequate drug bioavailability [22].

##### COVID-19 Vaccines

Very recently, in the race against the global COVID-19 pandemic, NPs proved again to be valuable drug carriers where various companies relied on nanotechnology for their vaccine design. For example, both the BioNTech/Pfizer partnership and Moderna developed an NP-based vaccine where mRNA, encoding subunits of the SARS-CoV-2 S protein, is encapsulated in lipid NPs for cytoplasmic delivery. The use of lipid NPs as carriers has several advantages: (1) intracellular delivery of mRNA is enhanced as a result of increased cellular uptake and facilitated endosomal escape, (2) the encapsulation prevents the degradation of the mRNA by nucleases in extracellular spaces, and allows chemical modification to further improve the RNA stability, (3) lipid NPs are composed of biocompatible materials suitable for human use and (4) GMP-grade lipid NPs are synthesized in a large scale. Additionally, the formulation in NPs also offers the possibility for codelivery of the virus antigens and adjuvants within the same NP reducing off-target side effects, fast adjuvant degradation and autoimmunity, and increasing the potency of vaccines at lower doses [23,24].

#### 2.1.2. Direct Nanoparticle-Toxicity

**Figure 2:**
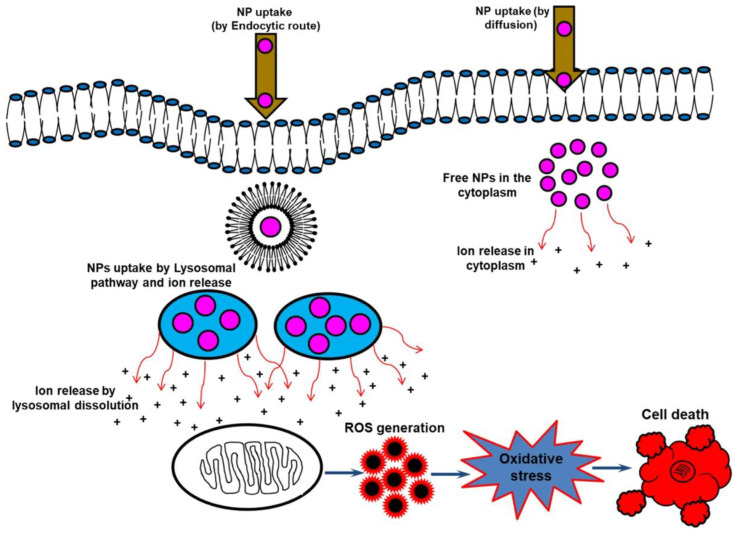
Schematic of the uptake and general toxicity mechanism of ion-releasing nanoparticles. NPs can enter cells by active internalization processes or endocytosis-free mechanisms. In case of uptake regulated by energy-dependent processes, NPs are rapidly confined in vesicular structures, endosomes, and finally in lysosomes. The acidic lysosomal pH triggers a lysosome-enhanced Trojan horse effect and enhanced release of the relatively toxic ions which subsequently results in elevated ROS levels, apoptosis, DNA and membrane damage. This figure has been reproduced with permission from Mir et al. [26] © 2020, Nature Publishing Group.

While NPs are often used as carriers for other therapeutic drugs, some NPs can be used by themselves as therapeutic agents to treat various diseases, including cancer. The exact mechanism of direct NP toxicity is still under debate; however, the release of ions is considered to be at least partly responsible for their toxic effects. The combination of an acidic environment and degradative capacity, both characteristics of endosomes, can induce the biotransformation of NPs and the subsequent release of ions responsible for intracellular toxicity. This cytotoxic process associated with ion-releasing NPs is also known as the “lysosome-enhanced Trojan horse effect” (Figure 2). Some examples of relatively toxic ions include Ag^+^, Cd^2+^, Fe^2+/3+^ Zn^2+^, Cu^2+^ and Au^1+/3+^ ions [25]. Although some NPs appear to exert specific toxicity against tumor cells, further investigation is needed to confirm these preliminary results [8].

##### Cancer Therapy

Recent results have highlighted the potential of direct toxicity of metal oxide NPs in tumor treatment as evidenced by their ability to efficiently kill therapy-resistant pancreatic tumor-initiating cells [27]. This may be driven, in part, by the physical size of the NPs, where it has been shown that they exceed the pore size of drug efflux pumps and hence remain inside their targeted cells [28]. Apart from a direct toxic effect, these NPs can also act to promote anticancer immune activation. As an example, Fe-doped CuO NPs were found to cause a transient reduction in tumor growth, along with neutrophil influx, inflammation, and an influx of cytotoxic T cells. When the NPs were combined with epacadostat, a compound tested in multiple clinical trials, that reverses IDO-1 immune suppression, this resulted in systemic immune activation and complete recovery from the tumor. Additional tumor rechallenge studies revealed that the effects were long-lasting, and the animals were protected from the tumor regrowth [29].

##### Antimicrobial Therapy 

Apart from its application in oncology, silver is one of the most potent natural antibiotics and has been used for many years to eradicate microorganisms. The use of nanosized silver particles with a large surface area enhances the contact between the particles and bacteria or viruses allowing for reducing the silver concentration without losing antibacterial efficacy [19].

##### Phototherapy

In addition to the release of toxic ions, the therapeutic effect of other, mainly inorganic, NPs can be influenced by externally applied stimuli such as in the case of NPs used as phototherapeutic agents themselves in phototherapy. Some materials that can be applied for thermal ablation of tumors include gold, iron oxide, and copper sulfide [19]. NanoTherm is an example of an NM already approved for hyperthermia treatment of brain tumors in the EU. This formulation, consisting of magnetic iron oxide NPs, is injected either directly into the tumor or into the resection cavity wall in case of tumor resection. After the insertion, a CT scan is obtained showing the distribution of the NPs in the tumor in detail. Subsequently, an externally applied, rapidly alternating magnetic field causes the NPs to generate heat, destroying the cancer cells or making them more sensitive to additional treatment approaches such as radiotherapy and/or chemotherapy. A thermometry catheter is used to monitor the temperature during the treatment. This approach allows eradication of remaining tumor cells in the resection cavity wall which is of utmost importance since these cells may be responsible for tumor recurrence. In addition, the surrounding healthy tissue is spared due to an aminosilane coating ensuring that the NPs remain at the site of application [30]. The major advantage of phototherapy is that it achieves selective tumor cell destruction minimizing damage to surrounding healthy tissue and thus reducing side effects [31]. Even though photosensitizers are also taken up by healthy cells, they preferentially end up and remain longer in diseased tissue as a result of the EPR effect [32]. Advances in imaging technology over the years have enabled precise anatomical identification of a tumor allowing the better focus of thermal ablation and preventing damage to healthy tissue and subsequent unwanted side effects. The safety profile of gold nanoshells, used as photothermal therapy (PTT) to treat prostate cancer, was evaluated in a clinical trial and revealed no indication of toxicity, lack of tolerance, or immunological effects during 6 months following particle infusion [33]. Another clinical trial using magnetic iron oxide NPs in combination with an alternating magnetic field to treat glioblastoma multiforme (GBM) also indicates that magnetic fluid hyperthermia does not cause any adverse effects on patients [20]. Although this type of therapy does not rely on therapeutic drugs, NPs themselves can cause adverse effects. Since NPs differ greatly from each other, each NP should be evaluated individually to balance the benefits against the potential risks [33]. Other advantages include the minimally invasive character of phototherapy, the absence of potential drug resistance occurrence, and the possibility of repeated treatment due to reduced toxicity [31,32]. Additionally, this approach also offers an alternative treatment in case the tumor cannot be removed by surgery [20].

##### Immunotherapy

In some cases, the toxic effect relies solely on the NP itself. Copaxone, a random copolymer consisting of L-glutamic acid, L-alanine, L-lysine, and L-tyrosine, is an example of a polymeric NM where the polymer acts as the therapeutic compound. This formulation was approved by the FDA in 1996 as an immunomodulator for multiple sclerosis [9].

### 2.2. Nanoparticles in Clinical Trials

NM is a rapidly expanding field where the number of clinical trials concerning nanosized materials has increased three-fold in just 3 years. Comparing the type of NMs already FDA-approved with those investigated in current clinical trials can give an idea of how the field is evolving. From the mid-1990s until 2015, FDA approvals for NPs mainly consisted of liposomal and polymeric formulations. In recent years, metallic and protein-based NPs represent higher percentages of the FDA approvals compared to previous periods. However, synthetic polymers are still often used as building blocks in this type of formulation [9].

Many of previously approved NPs are currently tested in clinical trials for additional indications. Since previously approved NPs were already validated for their safety and efficacy in humans, they are likely to obtain approval more easily when tested in trials for additional indications in comparison with newly developed systems [4]. In the case of anticancer NPs, these trials are mostly related to the approval of additional cancer types, combination therapy, or first-line therapy. Doxil, a chemotherapeutic seen as the gold standard of NMs, is currently tested in more than 160 clinical studies [4]. In addition, CRLX101 containing camptothecin, a DNA topoisomerase-I inhibitor, encapsulated in cyclodextrin-PEG copolymers NPs has already achieved phase I/II clinical trials in patients with rectal, ovarian, tubal, and peritoneal cancer. Other polymer-based NPs that already entered phase III clinical trials include NKTR-102 and Poliglumex, containing PEGylated etirinotecan and a polymer-drug conjugate of paclitaxel and polyglutamic acid, respectively. The trial of NKTR-102 showed that PEGylation, known to increase the circulation time of nanomaterials, enhanced the therapeutic response to etirinotecan by increasing the exposure time of tumor cells to the topoisomerase-I inhibitor [9].

As illustrated before, the application potential of NPs is very broad and definitely not limited to cancer. While trials in the oncology field still cover more than 50%, a clear shift can be observed towards other therapeutic areas including pain treatment, infection, and vaccination. Even studies on the clinical applications of NMs in new fields such as the nervous system, eye, and genetic diseases are arising which further indicates the great application potential of NPs [22]. For example, liposomal siRNA is currently in a clinical trial as gene therapy to knock down three different genes in the hepatitis B genome which consequently limits the antiviral resistance [34]. The focus of this type of therapy is more on treating a disease rather than solely reducing symptoms. In addition to the use of liposomes, which forms the basis in most clinical trials, formulations based on polymers and micelles are also investigated [4]. Feraheme, Venofer, and Ferinject, all clinically approved as iron replacement therapies, are investigated in multiple studies for approval in different clinical settings. Definity and SonoVue, already mentioned before as approved ultrasound contrast enhancers, remain also still active in clinical studies [4]. Additionally, iron oxide NPs for PET and/or MRI, such as Ferumoxytol, which was previously approved for iron replacement therapy, are at present abundantly investigated in clinical trials for imaging applications. This turn-around is probably a consequence of the discontinuation of all other iron-oxide imaging agents [4,22]. Several clinical trials also focus on NPs as delivery vehicles for contrast agents, such as 99Tc or 111In, for scintigraphy, SPECT, or PET analysis [22]. Apart from its use in diagnostics, NP-based imaging is also used in gene detection, protein analysis, cell tracking, and monitoring real-time therapeutic effects [6]. 

While many approved formulations are being tested for off-use applications, various novel strategies are also actively explored, further expanding the area of NP use in translational biomedicine. As such, the design of siRNA- or mRNA-containing NPs as gene therapy in cancer is broadly investigated in clinical studies [4]. The function of NPs in this approach is three-fold as they: (1) offer protection against enzymatic degradation by RNAses in the blood, (2) increase the accumulation of the content at the site of interest via the EPR effect, and (3) provide a cellular transport mechanism. Examples of this design approach include CALAA-01 and the SGT-53 system. CALAA-01 is a cyclodextrin-based, PEG-stabilized, and transferrin-targeting micellar system incorporating siRNA against the M2 subunit of ribonucleotide reductase. This system tends to achieve direct delivery to tumor cells and is regarded as the first targeted NP study in humans. The SGT-53 system, already successfully used to restore the function of human suppressor gene p53, is currently in phase ll clinical trials as a combination therapy with conventional chemotherapeutic agents [35,36]. It has also been shown that SGT-53 can limit the development of resistance to Temozolomide which often coincides with a poor prognosis in GBM [37].

Another innovative strategy that is widely explored is the packaging and delivery of chemotherapeutics by means of NPs. This design allows the use of highly toxic anticancer drugs that cannot be administered in the free drug form due to their toxicity profile. The VYXEOS/CPX-351 system is an example of a formulation that is developed to deliver a synergetic ratio of two anti-cancer drugs, cytarabine, and daunorubicin. Although the pharmacokinetic and off-site interaction profiles are unique characteristics of each drug, this particle encapsulation ensures that this exact drug ratio reaches the tumor [4]. The packaging of chemotherapeutics in NPs can also be used to obtain a higher tumor cell selectivity. For example, in the case of Cisplatin which is often used for cancer therapy but unfortunately also affects healthy organs as a free drug formulation. This issue can partly be resolved by using peptide-coated platinum NPs, which show a higher anticancer effectivity against HepG2 cancer cells in comparison with normal liver cells and other cancer cells in the treatment of hepatocellular carcinoma [38]. Two main factors contributing to the increased tumor cell selectivity of the platinum NPs include a high cellular uptake and an oxidative environment. Cancer cells show a higher glucose metabolism and express more glucose transporters on their surface compared to noncancerous cells. As a consequence, the cellular uptake of NPs in tumor cells can be further enhanced by adding a glucose unit to the peptides. The environment inside the cells also plays an important role since the cytotoxicity of Pt(II) ions is only revealed after the oxidation of Pt(0) to Pt(II). ROS, abundantly present in liver cancer cells, contribute to a high oxidation potential and increased selectivity of peptide-coated platinum NPs for these particular tumor cells. Additionally, the peptides are also important to ensure the stability of the NPs and prevent aggregation in water [38].

Other advanced approaches that introduce new concepts focus on chemical targeting and stimuli-responsive therapeutic release. The first targeting formulation for the delivery of siRNA used the transferrin receptor targeting protein and was proven to be successful in early clinical data. Other examples of chemical targeting include the conjugation with vitamin A to target stellate cells in the liver [39] and the conjugation with Herceptin, a HER2-targeted monoclonal antibody used to treat breast cancer [40]. Stimuli-responsive NP systems can be activated by external stimuli or biological conditions such as the presence of phospholipase A2 or binding with transferrin. While most of these formulations are inorganic systems used in the field of imaging, organic NPs can also be modified to respond to certain stimuli such as temperature or pH. In temperature-sensitive systems, the release of the component is induced by hyperthermia or ultrasound and for pH-sensitive systems, NPs can be designed to disintegrate only below or above a certain pH, releasing the active drug only in specific conditions or locations [4,38,41]. Incorporating such a triggering mechanism into NMs can improve the bioavailability of the encapsulated drug since drug release is not guaranteed by a mere accumulation of the nanocarriers in the tumor site. ThermoDox, a temperature-sensitive version of Doxil is currently investigated in clinical studies as a treatment for breast cancer and bone metastases. Based on this approach, the design of thermosensitive NPs containing both a therapeutic drug and a contrast agent can allow real-time monitoring of drug delivery and release and enables the prediction of therapeutic efficacy. Both substances are co-released in mild hyperthermia, induced by for instance high intensity focused ultrasound [41].

Taken together, the clinical progress, along with the ongoing developments made in preclinical studies involving NMs is further paving the way towards solidifying this important scientific research track into a robust clinically translatable discipline.

### 2.3. Limitations of Nanoparticles in the Clinic

Over the past few years, it has become clear that opinions about the progress of the NM field are quite diverse. Some consider that it has not lived up to the expectations and point at the very slow clinical translation of NMs, while others argue that the field has been undeniably successful and we should continue exploring it, yet without over-focusing on the nano aspect but with paying more attention to the medicine aspect [42,43]. The recent rapid and successful approval of the NM-based COVID-19 vaccines may have given the field a new impetus to head further.

So far, over 50 NMs have been approved by the FDA while more than 400 NM formulations are currently investigated in clinical trials [22]. The reason why the number of approved NPs is still limited can be explained by the multiple challenges NMs face upon entering the clinic. He et al. published an article in 2019 where they tried to unravel the high attrition rate and low translational success of cancer NMs specifically. While the success rate for phase 1 clinical trials is extremely high at 94%, this figure drops quickly to 48% and 14% for phase 2 and 3 clinical trials, respectively [44].

A first challenge that needs to be addressed, even before the development, concerns the commercial and practical feasibility where the improved patient benefit, the extent of the patient population as well as the treatment should be discussed.

A second critical bottleneck to consider is the understanding of NP interactions with biological components, which is closely related to bridging preclinical toxicology to clinical safety. Here, extensive characterization and safety assessment of newly designed NP systems are of utmost importance but are currently hindered by the lack of adequate analysis methods and standardized protocols. Moreover, the most conventional characterization assays often cannot be used to assess nanomaterials, and more complex approaches are required to evaluate how NP properties affect their safety and efficacy profiles.

A third concern is the limited reliability and validity of (murine) cancer models impeding the translation of preclinical efficacy to clinical outcome. Accurate animal models that precisely mimic human cancers, along with all tumor-specific properties such as mutation, proliferation, and metastasis, are required to address this issue. Additionally, often immune-deficient animals are used in preclinical trials which further complicates translation to patients with a properly functioning immune system. For anticancer NPs specifically, one should consider the fact that for certain tumor characteristics, such as tumor vascularization and stroma, substantial differences are observed among patients, within tumor tissue itself, and between different tumors in the same patient. Another aspect related to this challenge is the EPR effect which is controversially discussed in the recent literature. Some question whether the EPR effect might only apply to rodents while others describe substantial inter- and intravariability with respect to both the patient and the tumor type. Recent work by the Chan group observed that upon using so-called zombie mice, which are devoid of active transport mechanisms, NP transport from the bloodstream into the tumor was nearly completely absent, indicating that active transport mechanisms rather than the EPR effect play a major role in specific delivery of NPs to the tumor site [45]. A follow-up study by the same group, furthermore observed that tumor-associated endothelial vessels contain specialized endothelial cells, high in CD276 and PLVAP as biomarkers, that are the main drivers for transporting NPs from the bloodstream into the tumor microenvironment [46]. This also highlights another important issue, which lies in the overall poor delivery efficacy of NPs to solid tumors by intravenous administration. A meta-analysis of all NP delivery studies observed that on average, only 0.7% of the injected dose of NPs finally resided in the tumor [47]. Some authors made different calculations, more in line with classical pharmacokinetics, and concluded that the efficiency was better than presented in the original meta-analysis, but this re-analysis only calculated the ratio of NPs in the tumor and in the blood [48]. As NPs do not have a homogeneous distribution but are typically rapidly removed via the reticuloendothelial system (liver, spleen…), resulting in rapid blood clearance rates, these values could even exceed 100% and do not take into account any of the NPs already trapped elsewhere. This makes inter-study comparisons very difficult as good or bad tumor/blood ratios do not always correlate with the systemic NP distribution. Further advances in the field are however possible, where the efficiency of therapeutic moieties being delivered specifically to solid tumors by NPs was significantly enhanced by using a dual dosing strategy, where initially a high bolus of empty NPs is administered which saturates most phagocytic clearing cells, after which the therapeutic NPs were found to reach the tumor far more efficiently [49].

This brings us to a fourth challenge related to the clinical study design where it should be emphasized that proper patient selection, accurate consideration of therapeutic endpoints, and implementation of biomarkers predictive of therapeutic responses can limit clinical failures.

Additionally, there are some technical challenges in the production including difficulties in scale-up, the setup, and implementation of a manufacturing process according to Good Manufacturing Practice (GMP) standards, and the need for additional quality control assays on top of the conventional quality checks. The latter is essential to assess additional physico-chemical properties including particle size, size distribution and polydispersity, surface charge, drug loading, and drug release profile [1,22,44].

Last but not least, addressing the uncertainty and fragmentation in the regulatory framework. In this context, an important issue is the lack of an official, well-defined regulation for NMs specifically. At present, these formulations are treated in the same way as small conventional drugs and are required to meet the same criteria. However, as mentioned above, NMs possess additional physico-chemical properties that should be evaluated but for which currently no proper standards exist. Due to the wide range of possible modifications and the unique properties of different NMs, only a small number of standards are available at the moment, regardless of collective attempts to realize new standards. The assessment of several critical properties such as drug loading, particle stability in plasma and drug release kinetics, surface properties and surface interactions with the biological environment, and particle interactions with the immune system still lacks a clear and validated protocol.

To support the transition of nanotechnology into innovative medical applications, various initiatives were set up years ago including the European Technology Platform on Nanomedicine (ETPN), established in 2005, together with the European Commission (E.C.). The ETPN aims to accelerate the clinical adoption of NMs via the Nanomedicine Translation HUB providing assistance on three different levels namely custom mentoring, product characterization, and GMP manufacturing. These subjects are regulated by the HealthTech TAB (Translation Advisory Board), The European Nanomedicine Characterization Laboratory (EUNCL), and three medium-scale product lines (Nanofacturing, Nanopilot, and Maciviva), respectively. Additionally, the ETPN supervises an initiative, led by the National Institute of Standard and Technology (NIST) on the US side and the Joint Research Centre of the European Commission on the European side, which will further reduce the fragmentation of the current regulatory framework [22].

Another example of a project, established to support the development and clinical translation of nanotechnology, specifically for cancer applications, is the NCI Alliance for Nanotechnology in Cancer. This initiative was founded by the National Cancer Institute (NCI) in 2004 and covers four different programs including Centers of Cancer Nanotechnology Excellence (CCNEs), Nanotechnology Characterization Laboratory (NCL), Innovative Research in Cancer Nanotechnology, and Cancer Nanotechnology Training Centers [44,50].

While clearly various initiatives and platforms exist to facilitate the transfer of NMs from scientific design to clinical application, standardization and harmonization of the regulatory framework are urgently needed to further boost the clinical translation of NMs.

## 3. In Vivo Optical Imaging: Recent Developments and Limitations

Optical imaging refers to a wide variety of non-invasive techniques, using near-infrared (NIR) and visible light, to image organs, tissues, and even smaller structures including single cells in live preclinical animal models [51]. Contrast is generated through the detection of photons emitted by either luminescent or fluorescent sources. Whereas the visible light spectrum ranges from 400 to 700 nm, NIR light is defined by wavelengths ranging from 750 to 2500 nm. The latter can be subdivided into the first (NIR-I, 750–950 nm) and the second (NIR-II, 1000–1700 nm) NIR optical window [52,53]. Since autofluorescence, photon scattering, and absorption by biological tissues decreases with the increasing wavelength of the incident light, imaging in the second NIR window is preferred for in vivo deep-tissue imaging due to an improved penetration depth, resolution, and signal-to-background ratio (SBR) compared with visible and NIR regions below 900 nm [52,53,54]. Penetration depths up to sub-centimeter ranges can be achieved by imaging in the long end of the second NIR window (1500–1700 nm) which furthermore also completely eliminates tissue autofluorescence [55]. Although wavelengths beyond 1700 nm would even further reduce light scattering, imaging in this range is not preferred as water absorption increases and the sensitivity of indium gallium arsenide detectors, used for imaging in the second NIR window, is reduced [56].

Firefly luciferase is a commonly used luminescent probe and fluorescent markers are widely available, ranging from fluorophore-tagged antibodies to fluorescent proteins. A wide variety of optical imaging systems including endoscopy, spectroscopy, and optical coherence tomography are nowadays available for various biomedical applications. Compared to other imaging methods, optical imaging offers a number of advantages. One of the most important benefits is the high sensitivity, enabling the detection of low cell numbers and even single cells. For many interesting biomedical questions, it would be beneficial if small cell numbers could be easily visualized. At present, the latter is not possible since no suitable preclinical detection method exists yet. The currently used imaging methods for visualizing tumors such as ultrasound, MRI, PET, and CT lack high sensitivity and only show macroscopic effects over time. Therefore, these methods are not always suitable to evaluate, for example, NP uptake in tumors at the microscopic level [57,58]. The combination of commercially available fluorescent probes and the ability to genetically engineer cells or animals enables one to use optical imaging to study a wide spectrum of biomedical questions, which are not easily addressed by other imaging modalities. Optical imaging provides easy multiparametric imaging through spectral unmixing of different luminescent and/or fluorescent probes [59,60,61]. Preclinical systems based on optical imaging also allow high throughput analysis of animals making it possible to image up to 10 mice simultaneously. Since this imaging approach eliminates the need to sacrifice animals in order to obtain a functional read-out, it’s perfectly suited to monitor specific characteristics over time like, for example, the reduction of tumor volume in response to treatment [62]. Additionally, optical imaging systems are, relative to MRI or PET, far cheaper and thus more widespread. Data analysis is very straightforward and fast, enabling a wide variety of researchers with no knowledge of in vivo imaging to perform preclinical imaging studies all by themselves.

Bioluminescence, a specific type of luminescence, occurs naturally in nature where fireflies and other organisms chemically produce light to attract mates or prey during twilight. Bioluminescence imaging (BLI), a type of luminescence imaging often used in research, involves the detection of light, generated by the oxidation reaction between an enzyme and its substrate [63]. This approach offers an improved signal-to-noise ratio as external light source illumination is no longer required thus eliminating autofluorescence, in addition, cells and tissues show virtually no intrinsic bioluminescence [64]. The combination of firefly luciferase and D-luciferin is most commonly used in preclinical cancer research to monitor biological processes both in vitro and in vivo [62]. Since the maximum emission wavelength of D-luciferin in reaction with firefly luciferase is only 562 nm, functional readouts of probes located more deeply in tissues are hindered by the limited penetration depth of visible light. 

To date, optical imaging is applied in the field of oncology mainly to monitor tumor cell metabolism and viability in large solid tumors. Using the Aka-BLI technology, optical imaging could enable the detection and functional studies of major oncology-related questions that thus far have been very difficult to address due to the lack of sensitivity of the more commonly used methods mentioned above.

### 3.1. Recent Developments

Over the years, multiple strategies have been explored to expand the applicability of BLI. These efforts are manifold and focus on improving the expression levels of luciferases, increasing the bioavailability of the substrates by enhancing cell permeability and altering the tissue distribution, improving the efficiency of light emission, shifting the emission wavelength further into the red spectrum resulting in improved tissue penetration and on influencing the reporter system in order for the oxidation reaction to occur only in specific conditions [64]. Commonly used techniques include chemical modification of the enzyme and/or substrate, development of split luciferases and caged luciferin, bioluminescence resonance energy transfer (BRET), fluorescence by unbound excitation from luminescence (FUEL), bioluminescence assisted switching and fluorescence imaging (BASFI) and bioluminescent enzyme-induced electron transfer (BioLeT) [64].

**Figure 3:**
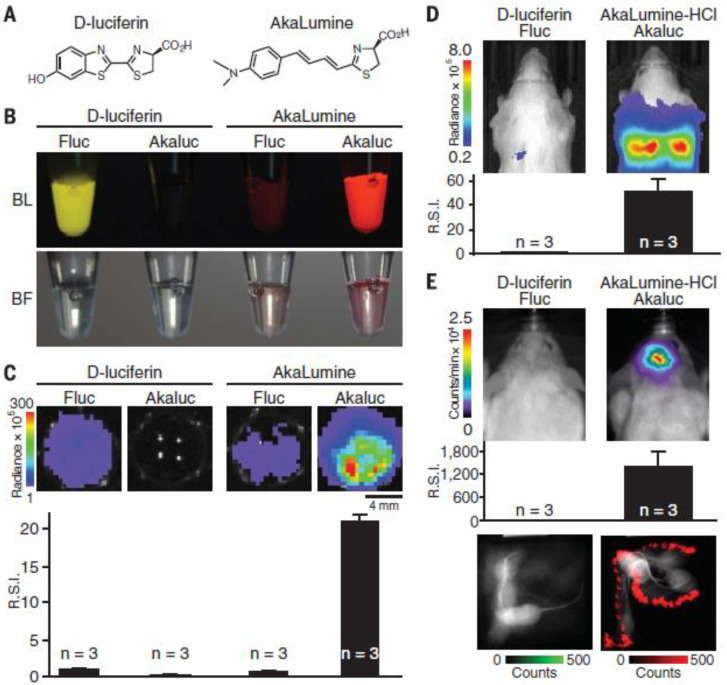
Performance of engineered AkaLumine/Akaluc versus natural D-luciferin/Fluc for in vitro and in vivo bioluminescence imaging. (**A**) Chemical structures of D-luciferin and AkaLumine. (**B**) BLI of four mixtures of the substrate (100 mM) and enzyme (2 mg/mL). Color images of solutions containing (from left to right) D-luciferin/Fluc, D-luciferin/Akaluc, AkaLumine/Fluc, and AkaLumine/Akaluc. BL, bioluminescence. BF, bright field. (**C**) Comparative BLI of cultured cells with the four substrate/enzyme combinations described in (**B**). HeLa cells expressing Fluc or Akaluc were treated with 250 mM D-luciferin or 250 mM AkaLumine and imaged using a cooled charge-coupled device (CCD) camera (1-min exposure time). Bioluminescence signals were quantified and normalized to that of the D-luciferin/Fluc system. Data are presented as mean ± SEM of three independent experiments. (**D**) Bioluminescence images of mice intravenously injected with 10^3^ HeLa cells expressing Fluc or Akaluc. Images were acquired using a cooled CCD camera (1-min exposure time). The AkaLumine-HCl/Akaluc signals were statistically compared to D-luciferin/Fluc signals. Data are presented as mean ± SEM of *n* = 3 mice. (**E**) Bioluminescence images of mice 2 weeks after viral infection for expression of Fluc and Akaluc in the right striatum. Immediately after substrate administration, anesthetized mice were imaged (top). The AkaLumine-HCl/Akaluc signals were statistically compared to D-luciferin/Fluc signals (middle). Data are presented as mean ± SEM of *n* = 3 mice. After intravenous injection with their respective substrates, mice were allowed to behave naturally in the arena (bottom). Bioluminescence and bright-field images (30-msec exposure time for each) were alternately acquired using an electron-multiplying CCD (EM-CCD) camera. An integrated image spanning 5 s is shown. Bioluminescence signals are shown in green (D-luciferin/Fluc) and red (AkaLumine-HCl/Akaluc). Bright-field signals are shown in black and white. Mice were injected with 100 to 200 mL of D-luciferin (100 mM) or AkaLumine-HCl (30 mM) (**D**,**E**). The color bars indicate the total bioluminescence radiance (photons/sec/cm^2^/sr) (**C**,**D**) and counts/min (**E**). R.S.I., relative signal intensity (**C**–**E**). This image has been reproduced with permission from Iwano et al. [65] © 2018, The American Association for the Advancement of Science.

A few years ago, Kuchimaru et al. developed a novel luminescent substrate, Akalumine-HCl emitting light more in the NIR field and thus enabling a deeper tissue penetration [63]. In contrast to D-luciferin, Akalumine-HCl is also tissue permeable allowing passage of the blood–brain barrier, and can therefore be applied to brain imaging [65]. In a follow-up study, they developed Akaluc, through directed evolution on firefly luciferase, which in combination with Akalumine-HCl resulted in a 1000-fold increase in brightness (Figure 3) [65]. This enabled them to rapidly visualize neurons in the brain of freely moving mice and to detect single cancer cells trapped within the lung vasculature upon intravenous administration. It should also be noted that the lung is one of the most challenging tissues to study with optical imaging due to the high scattering and absorption of light [63]. These results show that Aka-BLI is practically suitable for neuroscience to study neural circuitry and for other disciplines to monitor phenomena in naturally behaving animals [65]. Additionally, Iwano and colleagues also showed that oral administration of Akalumine-HCl resulted in the most persistent bioluminescence compared to intravenous and intraperitoneal administration. Such an approach of voluntary self-administration by offering unlimited oral access to a substrate solution enables the study of animals under natural conditions and evaluation of delicate behavioral changes [65].

Other examples of recently developed substrates that improve BLI systems are versatile and include among others the development of CycLuc1 [66], a substrate for codon-optimized luc2, two NanoLuc substrates hydrofumirazine and fluorofumirazine [67], and a luciferin analog with a 5-allyl-6-dimethylamino-2-naphthylethenyl moiety for reaction with Photinus pyralis luciferase and Akaluc [68]. Additionally, multiplexed BLI, where the target of interest expresses different luciferases interacting with different substrates, offers the possibility to simultaneously monitor multiple biological processes. Given that the biodistribution and uptake of sequentially administered substrates are different, complicating data analysis and contributing to bias, one can also use multiple luciferases interacting with the same substrate while generating sufficiently different emission peaks allowing spectral unmixing of the signals [64]. The latter approach is widely explored in research where recently multiple BLI systems were developed for simultaneous multicolor bioluminescence in vivo imaging. For example, Aswendt et al. described a model to discriminate two different human neural stem cell populations in the mouse brain based on multiplexed BLI [69]. Therefore, they combined a blue-shifted mutant (x5g) and a red-shifted mutant (x5r) of x5, a thermostable variant of wild-type firefly luciferase which also shows higher quantum yields. Substantial differences were observed in the in vivo spectra allowing us to calculate the contribution of each luciferase. Another strategy, explored by Stowe et al. was the development of infraluciferin, which shifts the emission wavelengths of two firefly luciferase mutants, FLuc_green and FLuc_red to 680 nm and 720 nm, respectively [70]. More recently, Zambito et al. developed a novel click beetle mutant, CBG2, which, in combination with CBR2 and the NH2-NpLH2 luciferin substrate, provides a system for dual-color deep tissue BLI where the enzymes emit light of 660 nm and 730 nm, respectively [71].

The use of a split luciferase system is a perfect approach to study protein–protein interactions where different parts of luciferase are attached to different proteins while caged luciferin can be applied to investigate enzymatic cleavage where one protein expresses the luciferase and the other the cleavage enzyme. BRET, FUEL, and BASFI are primarily used to study cellular proximity and protein–protein interactions by coupling the bioluminescent donor to one protein of interest and the fluorescent acceptor to the other [64].

While all these advances have been quite exciting for the field of NP distribution, the luminescence sources would mainly be useful in those systems where an active agent is delivered. For example, in a two-color luminescence system, one color can be expressed throughout the body (or only in the tumor), while the other color is not expressed until it is activated by recombinases. Using NPs loaded with mRNA or pDNA encoding for Cre recombinase, the distribution of the nanoformulations can be monitored over time, as well as the effect that the affected cells will have.

In view of NP biodistribution studies, various options can be selected. Typically, NPs can be labeled with NIR dyes (either by chemical conjugation or by staining with, for example, lipophilic dyes when using lipid NPs) [72]. Direct conjugation strategies are likely more reliable, as any dye that is embedded within a lipid bilayer by the hydrophobic effect can potentially leach out of the lipid bilayer and fuse into cell membranes. Apart from labeling the NP shell, another strategy lies in labeling the cargo within the NPs, such as mRNA or pDNA. While this is ideally suited for follow-up studies where, for example lysosomal release is studied, fluorescent labeling of genetic constructs can interfere with the function of the latter [73]. A third option also lies in exploiting the intrinsic fluorescent properties of (therapeutic) agents that can be loaded within the NPs. For example, indocyanine green can be efficiently used as a NIR contrast agent, but also a PDT moiety [74]. For chemotherapeutics, doxorubicin has also been shown to enable biodistribution studies [75], but this often requires end-stage experiments as the tissue penetration of Dox-induced light emission is not ideally suited for non-invasive whole body imaging.

One major problem for both fluorescence and luminescence monitoring of NP biodistribution is that neither method is intrinsically suited to provide any quantitative data on how many NPs have reached a certain tissue of interest relative to the number of NP administered. Recent advances in hardware have aided in overcoming this issue, where classical (2D or pseudo 3D) fluorescence imaging can now be performed by Fluorescence Emission Computed Tomography (FLECT). FLECT is a 3D optical tomography method that is ideally suited for detection of NIR fluorescence in deep tissue in living animals. The unique rotating gantry enable precise localization in three dimensions and absolute quantification of fluorescent signals, enabling highly detailed NP biodistribution studies [76].

### 3.2. Limitations

As highlighted above, optical imaging offers a number of advantages compared to other imaging techniques, however, some limitations still impede the use of common fluorescence and luminescence measurements. First of all, genetic engineering is required to obtain structures with fluorescent or luminescent properties and enable visualization by optical imaging. Various other methods exist to covalently and fluorescently label proteins of interest such as, for instance, the use of self-labeling enzymes, enzymes catalyzing the attachment of a fluorescent ligand to a protein, or small cell-permeable biarsenical dyes. Yet, they all vary in practical difficulty, and in some cases, fluorescent labeling is not even possible [77]. In addition, several strategies are employed to obtain NPs with fluorescent characteristics. Firstly, the fluorophores can be loaded on the NP surface or in the center of the NPs, the so-called “core-shell” structure [78,79]. NPs can also serve as quenchers by absorbing energy from the fluorescent dyes within NPs, keeping them in an “off” state during the circulation in the bloodstream [80,81]. After reaching the target site, the fluorescent dyes are released upon degradation of the NPs by specific enzymes, turning them into an “on” state. As an alternative, a separate quencher molecule can be added to the center of the NP [80]. The last design approach is based on fluorescence originating from an energy transfer between a donor and an acceptor molecule, also called Förster resonance energy transfer [6,81]. Another obstacle often faced with optical imaging, in general, is the limited penetration depth of light. However, since this parameter depends on the emission wavelength, deeper tissue penetration can be obtained by longer wavelengths, which also reduces cellular autofluorescence and scattering [77,82].

Some limitations of optical imaging specifically hinder the use of fluorescence such as real-time light excitation, which is essential for fluorescence imaging but can lead to phototoxic effects, mainly associated with long-term exposure of short wavelengths. The total dose of excitation light also influences this phototoxicity [76]. In addition, illumination inherently produces autofluorescence reducing the SBR and making it difficult to obtain reliable quantitative data [83,84]. In small animal imaging, this background fluorescence mainly originates from skin compounds, such as collagen, and food such as chlorophyll degradation products [82]. The emission spectrum of autofluorescence is usually very broad, which hinders the identification of the fluorescent signals of structures of interest. Since various approaches currently exist to eliminate or at least reduce this endogenous background fluorescence, this issue can easily be circumvented. The potential loss of fluorescence signal during tissue fixation is another problem that can fortunately easily be overcome by immunostaining with specific antibodies or FLAG epitopes. In addition, fluorescent labeling of a protein of interest can potentially impair the protein function and negatively influence the function of the cell [77]. To minimize this issue, one should strive for the lowest expression level of the fusion protein that still allows clear imaging. Some fluorescent proteins also show the behavior of blinking, meaning that the protein randomly switches between an “ON” and “OFF” state during continuous illumination [77]. Furthermore, many fluorescent dyes lack photostability, can bleach easily and have low quantum yields and are therefore also not very bright [77,85]. NPs can play a major role here, as they can serve as a pool of fluorescent dyes that are highly concentrated within a small, nanoscale volume and result in much higher brightness levels compared to free organic dyes. As such, quantum dots (QDs) offer a good alternative for organic dyes to label structures of interest due to their increased brightness and photostability [77]. Additionally, QDs have a long fluorescence lifetime and their emission wavelengths can be manipulated by changing the size [77].

Since bioluminescence is produced by an oxidation reaction between an enzyme and its substrate, the enzyme microenvironment and the biodistribution of the substrate play an important role [83]. Regarding luminescence, researchers often assume a direct correlation between the luciferase activity and the transcriptional activity of the reporter gene, resulting in a linear relationship between the BLI signal and the cell number. However, the bioluminescence reaction can be influenced by various endogenous and exogenous factors. The first disadvantage of BLI concerns signal quantification since the output, measured as relative light units, varies from one photon detector to another, contributing to great interlaboratory variability [86]. As it turns out, optical imaging also shows some drawbacks but most importantly, plenty of solutions and alternatives are available to overcome most of these issues.

## 4. Nanoparticles and Optical Imaging

In this section, we would like to address the question of why it is so interesting to combine optical imaging with NMs specifically and how they can improve one another. First, based on the abovementioned advantages of optical imaging and recent progress in this field, it is clear that this technique can be used to give more insight into the dynamic interactions between NPs and tissues at a subcellular level. The reason why NMs specifically, rather than other types of formulations, are interesting to combine with optical imaging can be explained by the fact that these materials show intrinsic or after simple modification, photon emission in the second NIR optical window. In this range, light shows less scattering and absorption by biological tissues and thus higher penetration depths can be reached.

As pointed out in Section 2.1, many clinical trials investigate the potential of NPs for the imaging and treatment of cancer. However, a better understanding of the biodistribution of NPs and the uptake process in tumor cells would be beneficial for further progress in the field of NM. Current studies, for example, often assume that the EPR effect plays a significant role in the distribution of NPs, although this process is still not fully understood [58].

### 4.1. Technical Progress

As described above, there is a wide range of preclinical imaging modalities available, including CT, MRI, PET, and SPECT, which can all be used to monitor the biodistribution of NPs. For CT, this would be limited to electron-dense NPs (e.g., gold) that are concentrated at high levels at the site of interest [87]. For MRI, magnetic NPs (e.g., Fe_x_O_y_) are required and quantification of the number of NPs is very challenging and not always possible. These methods are therefore less suited as general NP tracking tools, which further leaves PET and SPECT imaging as valuable options. Both with radiotracers, but in PET, the radiotracers must emit positrons, while for SPECT, they must emit gamma rays. Both are quite suitable for NP tracking and allow excellent quantification of the signal in the site of interest [88]. SPECT tracers typically have a longer half-life and therefore could be more optimally suited for longer-term studies, but they would still be limited in time to a few days [89]. Furthermore, the need for specialized and expensive equipment, along with the limitations and practical hurdles involved in working with radiotracers makes these methods less ideal. Optical imaging overcomes these problems with the broad range of fluorescent tracers and, in comparison, cheap and easy-to-use optical imaging systems that enable biodistribution studies. One major limitation for optical imaging however is the limited penetration of light, along with tissue scattering and the lack of precise 3D localization of the NPs. Classical 3D images are really 3D projections of 2D images and are therefore less suited to provide accurate 3D biodistribution studies and quantification of NPs. 

Recent technological progress has introduced more advanced systems that can overcome some of the hurdles mentioned above. For example, the lack of quantification in MRI has been overcome by the development of magnetic particle imaging (MPI), which enables rapid, long-term imaging of magnetic NPs in the entire body and can be accurately quantified [90]. For optical imaging, FLECT, as mentioned in Section 3.1, is ideally suited to provide accurate quantification of fluorescence and thus, provide a quantitative readout of NP biodistribution that can be monitored over time [91].

### 4.2. Biodistribution Studies

At present, the biodistribution of nanomaterials is most often studied by fluorescence microscopy of tissue slices using organic fluorescent dyes as optical probes. Important disadvantages of this approach include the inherent loss of the 3D structure of organs, lower SBRs due to autofluorescence, and the rapid photobleaching of fluorescent dyes reducing the possible observation time [85,92]. To overcome these drawbacks, various alternative methods are being investigated to study the biodistribution of NPs in vivo and to resolve issues that are until now unanswered. First, we will discuss several strategies to reduce the background signal, and afterward, we will highlight some optical imaging methods already used in the literature to study the biodistribution of NPs in vivo.

#### 4.2.1. Background Reduction by Cation Exchange

**Figure 4:**
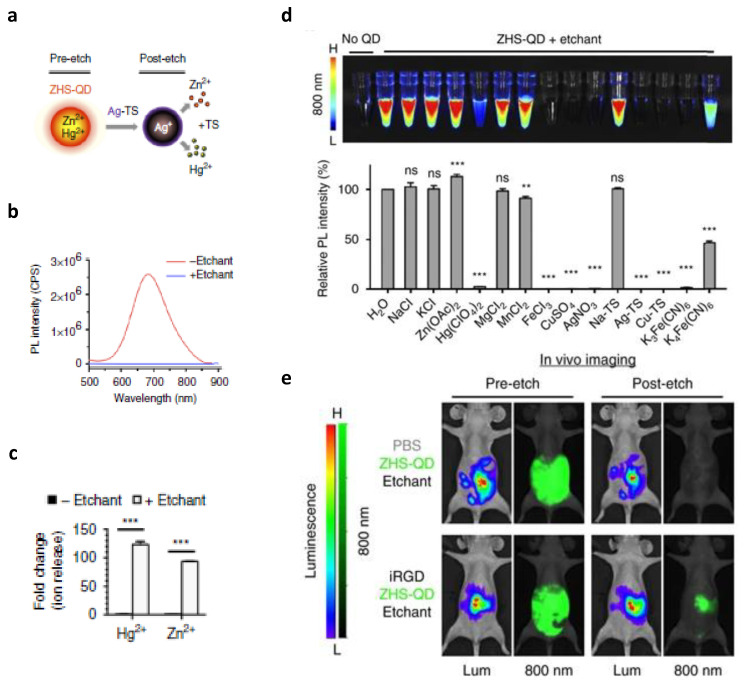
Characterization of etchable quantum dots containing zinc, mercury, selenide, and sulfide (ZHS-QDs). (**a**) Schematic of ZHS-QD etching. Ag-TS quenches ZHS-QDs by providing Ag^+^ in exchange for Zn^2+^ and Hg^2+^. (**b**) Photoluminescence spectra at 450 nm excitation of ZHS-QDs before and after treatment with [Ag(S_2_O_3_)_2_]^3−^, an etchant consisting of silver ions (Ag^+^) stabilized with thiosulfate (TS) (Ag-TS). (*n* = 6 per group; Scale bars, 50 nm). (**c**) Hg^2+^ and Zn^2+^ release from ZHS-QDs with and without Ag-TS treatment. Columns represent fold-over non-etched group. *n* = 3 per group. (**d**) In vitro etching of ZHS-QDs by various chemicals. NIR images (upper panel) and the emission intensity of the ZHS-QDs before and after etching (bottom panel, *n*  =  4 per group). Each column corresponds to the tube above. (**e**) Peritoneal tumor imaging with ZHS-QDs. Mice-bearing peritoneal tumors created with MKN45P-luc luciferase-positive human gastric cancer cells received an intraperitoneal co-injection of iRGD or PBS with ZHS-QDs. Intraperitoneal etchant (1x Ag-TS) was given 90 min later. *n*  =  3 per group. Statistics, Student’s *t*-test (**c**); error bars, SEM; ns, not significant; ** *p* < 0.01; *** *p* < 0.001. This image has been adapted with permission from Liu et al. [93] © 2017, Springer Nature.

The first approach that will be discussed here is the in vivo cation exchange in QDs, explored by Xangyou Liu et al. [93]. They designed a nanosystem in which background signals can be quenched, consequently increasing tumor specificity. QDs are very small semiconductor particles that often show fluorescent properties, making them suitable for optical imaging. In this study, photoluminescent QDs were modified with a membrane-impermeable etchant, serving as a cation donor. Upon exchange with internal cations, embedded in an anionic framework, photoluminescence characteristics are lost. This cation exchange can easily alter the elemental composition and crystal structure without influencing the anionic framework or the geometry of the QD core. Once the QDs reach the tumor site, the background signal is eliminated through the induction of cation exchange in the excess of QDs in the circulation. The cations released from these systemic QDs should be rapidly removed by renal clearance to assure minimal toxicity. In this study, QDs containing zinc (Zn^2+^), mercury (Hg^2+^), selenide (Se^2−^), and sulfide (S^2−^) were synthetized, and PEG coating was used to reduce MPS uptake. Silver ions stabilized with thiosulfate served as an etchant to quench the photoluminescence by exchanging Ag for Zn and Hg from the QDs (Figure 4).

This cation exchange has only a minor effect on the surface PEG layer. An increase in the QDs’ extinction coefficient was linked to enhanced fluorescence. The same etchant was able to successfully quench the photoluminescent signal of several QDs with different parameters showing the wide applicability of this quenching method. Additionally, this study revealed that QDs with an amphiphilic coating are resistant to the etching process by inhibiting the penetration of the cations. Simultaneous or subsequent administration of several QDs with different colors and etchable properties offers the possibility for multicolor and multiplex in vivo imaging. Moreover, this method allows in vivo imaging over short intervals since the QDs’ signal of repetitive injections can be effectively quenched by consecutive doses of the cation donor. Charging the surface of QDs can influence their uptake by specific cells and tissues, maintaining a signal in a certain organ after etching. In this study, it was shown that a positive charge increased the uptake in Kupffer cells. Data also showed that intravenous injection of iRGD (CRGDK/RGPD/EC) peptide enhanced the tumor signal. It is known that iRGD peptide increases the extravasation and endocytosis of the peptide and bystander molecules, a process depending on the tumor type. The bystander effect can be exploited to actively deliver co-injected QDs to tumors. According to this study, etching is limited to the extracellular space as QDs, internalized by cells in the extravascular tumor tissue, stay intact after injection of the cation donor. Another type of QDs, without Hg, was also tested to avoid any potential toxicity of Hg in vivo. These QDs, containing Zn, Ag, Se, and S, were also coated with PEG and showed similar etchable properties. Both of these ionic etching systems based on QDs, with and without Hg and combined with iRGD, provide a promising method for tumor-specific in vivo imaging [93].

#### 4.2.2. Background Reduction by Photobleaching

**Figure 5:**
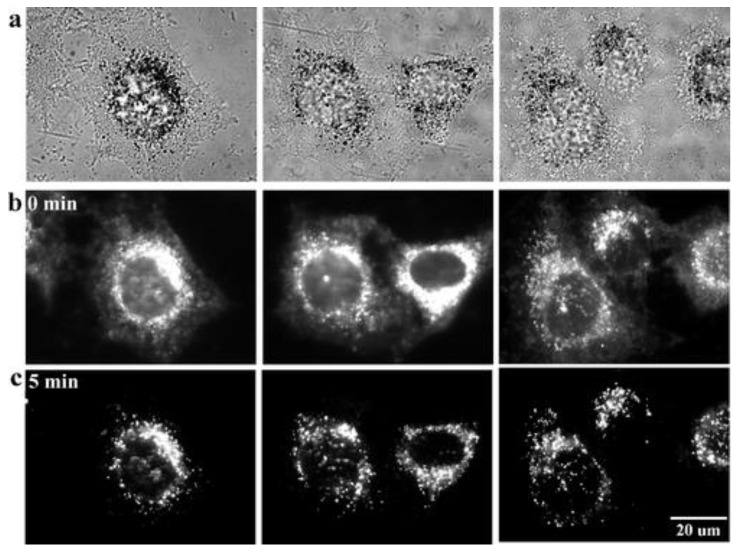
Nonbleaching fluorescence of gold nanoparticles. (**a**) Bright-field images and (**b**,**c**) fluorescence images of HeLa cells incubated with 38 nm colloidal gold NPs (**b**) before and (**c**) after 5 min photobleaching of cellular autofluorescence. The other two images in the same row for each imaging condition are shown to test reproducibility. Exposure time: 5 s. This image has been reproduced with permission from He et al. [85] © 2008, American Chemical Society.

Another imaging approach that is explored to study the biodistribution of NPs is based on nonbleaching fluorescence which furthermore extends the observation time. Autofluorescence, stemming from tissues and cellular components is easily photobleached, meaning that the fluorescent characteristics are lost due to irreversible modifications after a certain period of strong light illumination. Many currently used fluorophores also show the behavior of photobleaching, limiting the observation time and making it difficult to eliminate the background autofluorescence. Lately, QDs have been increasingly used as optical probes but, although they show better photostability, illumination still causes some decomposition. In addition, these QDs often contain heavy metals rendering them inappropriate for in vitro and in vivo analysis. As an alternative, colloidal gold NPs (GNP), possessing the beneficial characteristic of anti-photobleaching, are evaluated as fluorescent probes for optical imaging (Figure 5). In addition to the fact that their synthesis is very straightforward, they also show great biocompatibility and they can be easily conjugated with other components. Furthermore, data show that the emission intensity can be improved by increasing the particle size while at the same time keeping the fluorescent emission wavelength constant. These GNPs are taken up by endocytosis and accumulate in the cytoplasm. Cell morphology did not seem to be affected after continuous illumination for 10 min. Conjugation with epidermal growth factor receptor (EGFR) antibodies has been shown to successfully target HeLa cells, which exhibit a significantly increased EGFR expression. This newly developed cellular imaging method, using GNP as fluorescent probes, showed promising results in vitro and offers great potential for applications in cancer diagnostics and therapy, single particle tracking, targeted drug delivery, and immunoassays [85].

#### 4.2.3. Background Reduction by Persistent Luminescence

**Figure 6:**
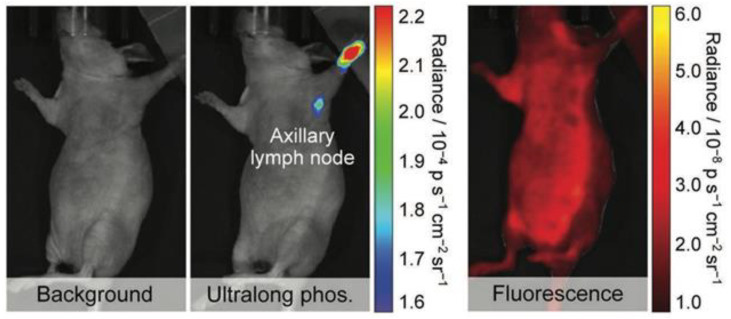
In vivo optical imaging of organic semiconducting nanoparticles (OSNs). Ultralong phosphorescence and fluorescence imaging of lymph node in living mice 1 h after the intradermal injection of OSN1-T (1.6 × 10^−6^ M) into the forepaw of mice. This image has been reproduced with permission from Zhen et al. [94] © 2017, Wiley-VCH.

Persistent luminescence, also called afterglow, is another method evaluated to study the biodistribution of NPs. The mechanism behind this phenomenon, where luminescence can last for some time after the cessation of light excitation, is based on the slow release of photons from energy traps in the materials caused by thermal stimulation. Afterglow is known to be a characteristic of mainly inorganic NPs often containing rare earth (RE) heavy metals such as europium and chromium. This chemical composition raises questions related to the safety of these NPs and therefore Xu Zhen et al. designed organic semiconducting NPs, using a top-down approach, for in vivo afterglow imaging [94]. In this approach, imaging sensitivity and specificity are increased since tissue autofluorescence is eliminated. Their study revealed that phosphorescence can be prolonged by maximizing the formation of H-aggregates within NPs. These aggregates stabilize the triplet excited states, and their formation is enhanced by stronger molecular packaging. Good water solubility was accomplished by adding an amphiphilic triblock copolymer. The possibility to reactivate the luminescence signal turns these organic semiconducting NPs into valuable material for longitudinal imaging. Moreover, this form of phosphorescence is, in contrast to conventional phosphorescence, not quenched by oxygen and makes it particularly suitable for in vivo imaging where oxygen is abundantly present. Xu Zhen et al. also performed an experiment showing the applicability of the organic semiconducting NPs in lymph node mapping in live mice (Figure 6) [94]. The latter is often carried out to determine whether cancer cells have spread from the original tumor to nearby lymph nodes and to guide surgical resection of tumor tissue. The axillary lymph node could be clearly imaged after intradermal injection of the organic semiconducting NPs into the forepaw while the contrast with fluorescence imaging is much lower, making it impossible to distinguish lymph nodes and normal tissue. In this study, the same top-down method was used to prolong the phosphorescence of different semi-conducting dyes showing its suitability for other organic systems [83,84,94].

**Figure 7:**
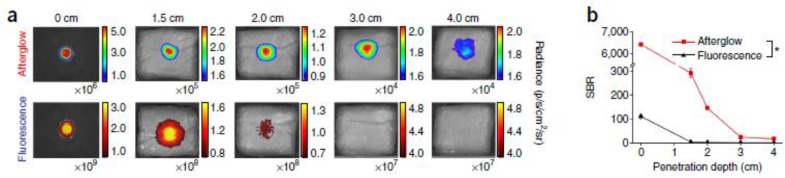
Tissue-penetration study of NIR afterglow luminescence. (**a**) Afterglow luminescence (upper panel) and fluorescence (lower panel) imaging of semiconducting polymer NPs (SPN) doped with a ^1^O_2_ sensitizer, silicon 2,3-naphthalocyanine bis(trihexylsilyloxide) (NCBS) to obtain SPNs with a 5% weight percentage (*w*/*w*) of NCBS. The thickness of the chicken tissues is defined above the images. (**b**) Signal-to-background ratios (SBRs) for afterglow luminescence and fluorescence of SPNs containing 5% (*w*/*w*) NCBS as a function of tissue depth. *Statistically significant difference between afterglow and fluorescence intensities in SBRs through chicken tissues at 4 cm (*n* = 3, unpaired *t*-test (two-tailed), t = 95.47, df = 4, * *p* < 0.0001). This image has been adapted with permission from Miao et al. [83] © 2017, Springer Nature.

Since most existing persistent luminescent agents are not biodegradable and raise long-term concerns as mentioned before, Qingqing Miao et al. designed two different biodegradable organic semiconducting polymers NPs (SPNs) as afterglow luminescent probes using biologically benign ingredients to ensure biocompatibility and safety [83] This elemental composition shows good enzymatic degradation and clearance highlighting its translational value. Additionally, SPNs can be adjusted to be used for fluorescence, chemiluminescence, bioluminescence, PTT, and other approaches. The design of the first SPNs required a precipitation step with an amphiphilic polymer to obtain water-soluble NPs and resulted in relatively large NPs which could potentially impede the biodistribution. To solve this problem, Chen Xie et al. designed a second SPN consisting of amphiphilic polymers with a hydrophobic poly(p-phenylenevinylene) (PPV) backbone and a hydrophilic PEG part allowing the self-assembly into small NPs in an aqueous environment [84] Upon irradiation of the PPV-based SPNs, unstable chemical units are formed which spontaneously and slowly break down releasing photons and generate afterglow luminescence. The tissue penetration study clearly shows that the SBR for afterglow was higher for all thicknesses of chicken tissue compared to fluorescence (Figure 7). These results can be explained by the low background noise associated with afterglow [83]. 

Since the decomposition is a slow and gradual process, it is possible to repeatedly recharge the afterglow enabling long-term in vivo imaging. In addition, afterglow luminescence depends on the temperature and the oxygen levels and can be modulated by, for example, adding an ^1^O_2_ scavenger. It was shown that the afterglow intensity increased with higher temperatures. Such temperature sensitivity highlights the applicability of afterglow imaging to monitor the temperature during PTT. Since afterglow is sensitive to oxygen, this imaging approach can be used to monitor oxygen levels in vivo and differentiate between normoxia and hypoxia, associated with the tumor environment. They also discovered that the afterglow intensity can be increased, and both the pre-irradiation and emission wavelengths can be shifted towards the NIR region by doping the SPNs with an ^1^O_2_ sensitizer. Scavenging or enhancing the generation of ^1^O_2_ can, respectively, decrease or increase the afterglow brightness. The same SPNs were used to design activatable probes to monitor drug-induced hepatotoxicity in vivo. The facile structural alteration of SPNs provides an excellent approach to developing other activatable probes to monitor the progression of various pathological diseases. Since the second type of SPNs are smaller and have a higher PEG density, they show an increased accumulation in the tumor. These SPNs have a very high SBR allowing detection of smaller tumors and at earlier time points post-injection compared to the first type of SPNs. To conclude, NIR afterglow of SPNs, doped with an ^1^O_2_ sensitizer, enables faster and more sensitive lymph node and tumor imaging compared to NIR fluorescence imaging. The structural flexibility of SPNs makes these agents perfectly suitable as a base for the development of various afterglow probes for deep-tissue imaging [83,84].

Metal-enhanced and nonbleaching fluorescence, as well as the development of phosphorescent NPs, emphasize the possibility of designing NPs with specific characteristics, allowing longer in vivo visualization of NPs with increased sensitivity. In combination with optical imaging techniques, these approaches show great potential for biological applications.

#### 4.2.4. Background Reduction by Exploiting the Second Optical Window

As mentioned in Section 3, imaging in the second NIR optical window improves the tissue penetration depth and offers high spatial and temporal resolution, unattainable and/or far exceeding those achieved by other imaging techniques, as light scattering, absorption, and autofluorescence are reduced at higher wavelengths [56,95]. To date, several types of NIR-II fluorescent probes have been developed including small molecules, carbon nanotubes (CNTs), semiconductor QDs, lanthanide-doped NPs, conjugated polymers, organic dyes, and organic dye-based NPs [96]. While some of these show emission in the long end of the second NIR window, the brightness is still limited, and concerns related to biocompatibility and toxicity hamper the clinical translation [55]. Hence, more research is needed to exploit this favorable spectral range and expand the in vivo fluorescence imaging applications.

In this context, interesting NP formulations worth mentioning are the CNTs, formed by wrapping a single or multiple graphene sheets resulting in needle-like shaped, single-walled (SWCNTs) and multi-walled (MWCNTs) CNTs, respectively. Whereas the diameter of SWCNTs is mostly only a few nm, it can go up to 100 nm for MWCNTs. CNTs exhibit high specific surface areas due to their small size and high aspect ratios and are endowed with unique mechanical, electrical, and optical properties [97,98,99]. They show intrinsic fluorescence with excitation in the first NIR optical window and emission in the second NIR optical window allowing deeper tissue penetration. Owing to their hollow interior, CNTs are ideal candidates as carriers for hydrophobic drugs and furthermore, they show the exceptional capacity to easily penetrate cell membranes and enter cells making them perfect candidates for intracellular delivery of not only drugs but genes and proteins as well. CNTs are particularly interesting as mediators and carriers for cancer therapy where they have already been used as nanocarriers for anticancer drugs such as doxorubicin and paclitaxel [97].

Hong et al. designed SWCNTs for in vivo real-time epifluorescence imaging of mouse hindlimb vasculature providing both high spatial and temporal resolution with tissue penetration depths of 1–3 mm [95]. Where current techniques are suboptimal as a single modality to obtain both anatomic and hemodynamic information, these NIR-II emissive SWCNT provide a multifunctional imaging method allowing to simultaneously differentiate arterials and venous vessels as well as to accurately quantify blood velocity using a dynamic contrast principal component analysis (PCA) approach. The latter can be interesting to, for example, evaluate the degree of occlusion due to ischemia. While micro-CT or MRI can be successfully applied to resolve arterial and venous vessels anatomically down to 100 µm, these techniques suffer from long scanning and post-processing times and are less suited to study vascular hemodynamics. Meanwhile, micro-ultrasonography is usually applied to obtain hemodynamic data but for this technique, the spatial resolution attenuates with increasing penetration depths. Where the NIR-II signals of the SWCNTs allowed us to image smaller, higher-order branches of blood vessels at higher magnification, imaging in the NIR-I window using IRDye-800 failed to measure vessel diameter. NIR-II imaging results in a penetration depth exceeding 5 mm as even the aorta could be clearly visualized with the mouse laying on its back.

SWCNTs were also developed by Diao et al. using the laser vaporization (LV) method which resulted in CNTs with larger average diameters and higher fluorescence in the 1500–1700 nm region compared to the widely used high-pressure carbon monoxide conversion (HiPCO) SWCNTs [53]. High-magnification microscopic vessel imaging of both mouse hindlimb and brain resulted in improved feature sharpness and increased SBRs when imaged in the 1500–1700 nm region in comparison with the 1300–1400 nm region. Video-rate fluorescence imaging of blood vessels in the mouse hindlimb in the long end of the second NIR window enabled simultaneous mapping of blood flow velocities over a broad range of 1–20 mm s^−1^ with spatially resolved individual vessels [53]. Video-rate fluorescence imaging was also performed for in vivo tumor imaging where the distribution of intravenously injected LV SWCNTs to the lungs, and afterward, to the major organs and vascular structures surrounding the tumor could be clearly monitored.

**Figure 8:**
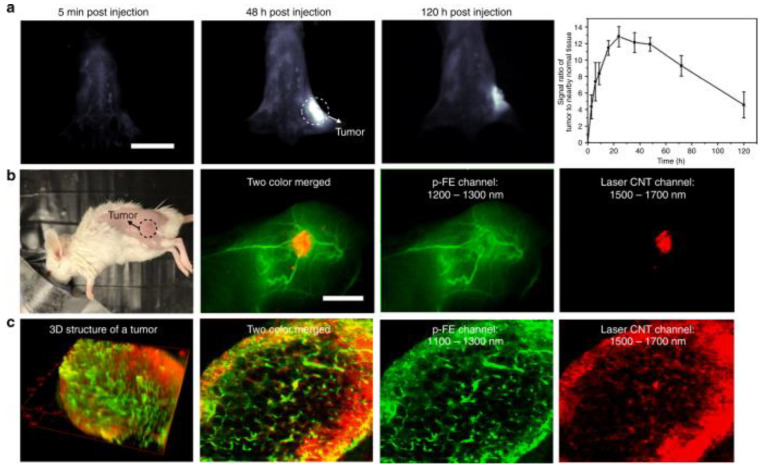
Two-color fluorescence imaging of a tumor in the NIR-II window. (**a**) Wide-field fluorescence imaging of a mouse inoculated with a 4T1 tumor through collection of fluorescence emitted from a bright organic nanofluorophore (p-FE) above 1300 nm with exposure time of 5 ms and variation of T/NT signal ratio as a function of time (n  =  3). (**b**) High-magnification fluorescence imaging of a mouse inoculated with a 4T1 tumor using two colors in the NIR-II window. (**c**) Confocal imaging of a tumor using two colors in the NIR-II window. Area: 740 µm  × 740 µm × 220 µm, step size: 2 µm along x and y directions, 5.4 µm along z direction. Laser power ~30 mW, PMT voltage: 500 V for p-FE channel and 600 V for laser CNT channel, scanning speed 15 min/frame. Pin hole: 150 µm for p-FE and 300 µm for laser CNT channel. Wavelength range: 1100–1300 nm for p-FE channel and 1500–1700 nm for laser CNT channel. This image has been reproduced with permission from Wan et al. [100] **©** 2018, Nature Research.

Wan et al. designed a bright organic nanofluorophore (named p-FE) by exploiting the hydrophobic pocket of an amphiphilic polymer to encapsulate an organic NIR-II dye with a fluorescence peak of 1010 nm [100]. This approach preserves the high quantum yield in aqueous environments by preventing aggregation and fluorescence quenching as well as endowing the probe with aqueous solubility. They successfully performed in vivo real-time tracking and velocity quantification of fast-moving blood flows in the cerebrovasculature of mice through intact scalp and skull. Additionally, the probe enabled one-photon 3D confocal layer-by-layer imaging of fixed mouse brain tissue at imaging depths up to ~1.3 mm and sub-10 µm spatial resolution [100]. In vivo tumor imaging revealed a remarkable tumor-to-normal tissue (T/NT) ratio of ~12 and in vivo two-color imaging allowed simultaneous imaging of tumor tissue and vasculatures by CNT and their own organic nanofluorophore, respectively (Figure 8) [100].

Zhou et al. also developed a kind of organic NPs, namely ultrabright polymer dots (Pdots) with an emission maximum centered near 1020 nm for real-time detection of metastatic ovarian cancer [96]. The probe design is based on the self-assembly process in water between NIR-II emissive aggregation-induced emission luminogens and amphiphilic poly (styrene)-graft-poly(ethylene glycol) and showed significant improvement in single particle brightness compared with other NIR-II fluorescent NMs including QDs and lanthanide-based probes. To evaluate the penetration depth, well plates were covered with pork tissue of different thicknesses revealing a penetration depth of up to 7.5 mm [96]. Additionally, the probes were further modified with ovarian cancer targeting GnRH peptide to enhance tumor cell affinity. In vivo experiments showed the successful use of whole-body vessel imaging, tumor imaging, and metastatic tumor imaging. Subcutaneous tumor imaging revealed more rapid tumor accumulation, longer tumor retention time, and a higher T/NT ratio of the targeted probe compared to the non-targeted. Peritoneal and lymphatic metastasis could be clearly visualized in vivo by the GnRH-targeting Pdots. Guided surgery using the targeting probes enabled the precise resection of metastatic foci with a diameter down to ~2 mm whereas the small peritoneal metastatic lesions could not be identified with the non-targeted Pdots [96]. Time-dependent fluorescence imaging was also performed to further study the biodistribution of the Pdots and revealed clear signals in the main organs deep in the tissue including the liver, spleen, lung, and kidneys.

Apart from CNTs and organic compounds, inorganic NP-based fluorescent probes such as QDs, RE NMs, and down-conversion NPs (DCNPs) are also promising candidates for enabling imaging in the second optical window [54]. They exhibit some unique properties compared with other fluorophores emitting in this wavelength range. For example, their fluorescence emission wavelengths can be easily modified by altering their size and composition. Furthermore, QDs specifically show broad excitation yet narrow and sharp emission spectrum and single excitation/multiple emission, ideal for multiplex imaging. While their large specific surface area opens possibilities for modification and conjugation, they can also be readily modified to obtain additional properties enabling multimodal imaging with for example MRI and CT. Inorganic NP-based fluorescent probes have already been shown to be successful for a variety of applications such as nonspecific imaging including imaging of vasculatures and the lymphatic systems, as well as specific imaging and light sheet microscopy (LSM) [54]. When associated with PCA, arteries, veins, and organs deeply under the skin can be identified. Even cerebrovascular imaging and quantification of cerebral blood flow velocity without craniotomy is at present possible using lead sulfide (PbS) QDs, InAs QDs, and DCNPs. The use of PbS and Er DCNPs allowed in vivo identification of tumors and metastatic lesions as small as ~4 and ~3 mm, respectively [54]. Recently, Huang et al. reported the application of Ag_2_S QDs to track the location, survival, and osteogenic differentiation of transplanted stem cells in a mouse model over a period of 30 days using a multiplexed imaging strategy [54]. While most LSM instruments employ excitation and emission in the visible spectrum, Dai et al. combined this technique with PbS/cadmium sulfide (CdS) QDs emitting in the second optical window thereby enabling in vivo 3D imaging of a traumatic brain injury model without invasive surgery. By conjugating the QDs with antibodies targeting specific tumor features, they were able to monitor T-cell dynamics in the tumor microcirculation and to detect programmed cell death protein 1 and programmed death-ligand 1 (PD-L1) in tumors with cellular resolution.

Zhang et al. also developed QDs with a core–shell structure consisting of a PbS core surrounded by a CdS shell [56]. Common problems of PbS QDs include surface oxidation and a massive decrease in the fluorescence intensity and photostability upon phase transfer to an aqueous environment needed for biocompatibility. The outer shell of CdS protects the PbS core by effectively preventing its chemical and photochemical degradation and by contributing to its surface passivation and thus maintaining high fluorescence intensity upon transferring to aqueous solutions. The resulting QDs emit light at ~1600 nm and allowed fast real-time imaging of blood flows in the mouse vasculature [56]. Moreover, they enabled through-skin in vivo 3D one-photon confocal imaging of tumor vasculature with a depth of ~1.2 mm providing an excellent alternative to multiphoton confocal imaging which involves the need to surgically implant an optical window. When used for in vivo non-invasive imaging of a mouse tumor, a superior T/NT ratio, reaching 32.6 as the highest, was achieved [56].

Similar PbS/CdS core–shell QDs were designed by Jeong et al. and applied for multiplexed in vivo tumor imaging in the second NIR window [101]. They prepared two polymer-encapsulated QD probes emitting at 1080 nm and 1280 nm showing negligible spectral overlap and enabling simultaneous monitoring of several molecular targets and complex biological processes. To investigate tumor-targeting ability, the 1080-QDs were conjugated with folic acid (FA) to target folate receptors (FR) which are overexpressed in some human cancer cells. Co-incubation of the FA-QDs with two different FR-positive human cancer cell lines, Hela and MCF-7, revealed bright fluorescence whereas no noticeable signal was observed for the non-targeting control [101]. The tumor-targeting ability was also compared in a mouse tumor model injected with FR-positive Hela cells in the right flank. Intravenous injection of a mixture of the targeting and non-targeting QDs allowed real-time discrimination of both probes and quantification of T/NT ratios.

**Figure 9:**
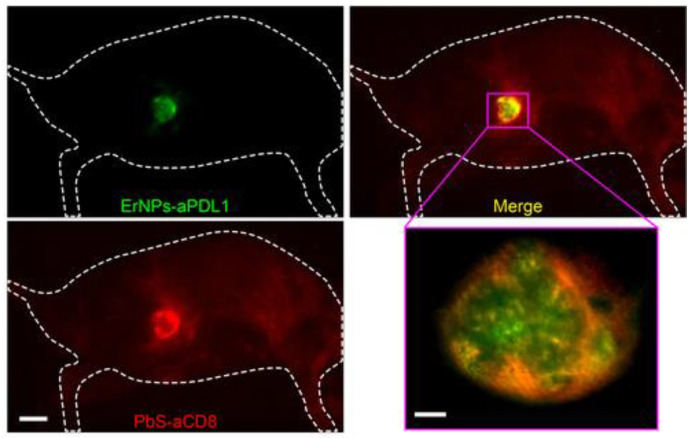
In vivo two-plex NIR-IIb molecular imaging of immune responses using ErNPs-aPDL1 and PbS-aCD8 at the same ~1600 nm emission range. Two-plex molecular imaging (upper right) of a CT-26 tumor mouse at 24 h post intravenous injection of mixed ErNPs-aPDL1 (green color, upper left) and PbS-aCD8 (red color, lower left). Scale bar: 5 mm. The zoomed-in high-magnification two-plex image (lower right) outlined the CT-26 tumor with micrometer image resolution (scale bar: 500 μm). This image has been adapted with permission from Zhong et al. [55] © 2019, Nature Publishing Group.

Zhong et al. developed novel Zn-doped cubic-phase (χ-phase) erbium-based RE NPs (ErNPs) with a core–shell structure showing downconversion luminescence at ~1600 nm, enabling imaging with subcentimeter tissue penetration and micrometer resolution [55]. The tissue biodistribution, pharmacokinetics, and excretion could be easily investigated by monitoring the luminescence signals in the major organs revealing a biliary excretion pathway. They successfully performed real-time imaging of mouse cerebrovasculature through intact scalp and skull as well as ultrafast hindlimb vasculature imaging. Additionally, they designed a two-plex imaging model to dynamically image and assess the response to cancer immunotherapy by simultaneously tracking PD-L1 and CD8+ cytotoxic T lymphocytes (CTLs) in vivo (Figure 9). By blocking the PD pathway, anti-PD-L1 antibody treatment prevents cancer immune evasion and induces antitumor immunity where activated CTLs migrate from lymphoid organs rich in immune cells into the tumor and kill cancer cells by inducing apoptosis. The ErNPs targeting PD-L1 showed T/NT ratios of ~40 whereas a much lower ratio of ~11 was obtained for the free non-targeted ErNPs as a result of the EPR-effect [55]. While both PD-L1 and CD8+ probes emit in the 1500–1700 nm range, signals were easily distinguished as the ErNPs exhibit a long luminescence lifetime in the range of milliseconds, and QDs show short-lived microsecond fluorescence. Where the PD-L1 signals were evenly distributed within the tumor, the CD8+ signals were higher around the borders of the tumor indicating the infiltration of activated CD8+ CTLs primarily from the periphery [55]. This model allows us to simultaneously visualize the biodistribution of tumor and immune cells, both emitting at the long end of the second NIR window, and could possibly be combined with the currently applied biopsy-based diagnostic assays in the clinic. Additionally, the ErNPs-aPD-L1 show great potential as a theranostic agent providing both PD-L1 imaging and immunotherapy.

While NPs emitting in de second optical window clearly show promising potential to improve fluorescence imaging, some challenges remain difficult to resolve [54]. For example, finding the right balance between the circulation time and excretion rate, developing multicolor probes generating sufficiently different emission peaks to allow multiplexed imaging, designing heavy metal-free NIR-II fluorescent probes to avoid toxicity issues, and combining NIR-II guided diagnosis and therapy through the development of a multifunctional NIR-II probe.

#### 4.2.5. High-Resolution Imaging by Intravital Microscopy

**Figure 10:**
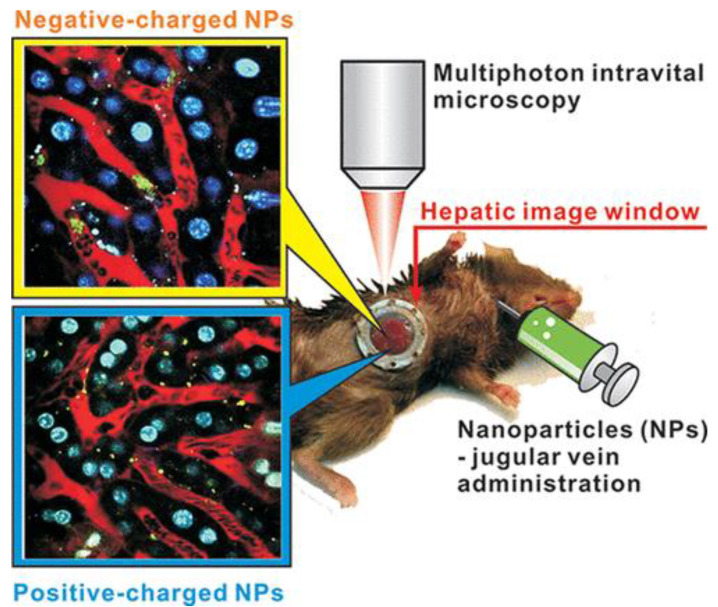
Multiphoton intravital microscopy. A hepatic imaging window was implanted to visualize the sub-hepatic distribution of different surface-charged NPs in real time at subcellular resolution using multiphoton imaging. This image has been reproduced with permission from Cheng et al. [104] © 2012, American Chemical Society.

Intravital microscopy (IVM), which generates high-resolution images using lasers and photodetectors, is often used to monitor biological processes in vivo. This imaging method provides structural and functional information with subcellular resolution and allows real-time monitoring of NP biodistribution. IVM has already successfully been used to study tumor-specific delivery and accumulation of NPs [102], to predict NP delivery efficacy [103], and to visualize the interactions of NPs with various cell types [104]. In addition, this method allows us to dynamically study NP interactions with subcellular resolution at various time points instead of at a single time point, as is the case with for example histology [58]. This important feature makes it also an excellent method to longitudinally monitor neovascularization [105], disease progression [106], and treatment outcome [107]. Choi-Fong Cho and colleagues already proved the efficiency of IVM to evaluate NP uptake in tumors using human tumor xenografts in a modified, shell-less chicken embryo model. Tumor cells were implanted into the chorioallantoic membrane and different NP sizes, and formulations were tested. They were able to follow various processes involved in the biodistribution of fluorescently labeled NPs such as the circulation throughout the body, the subsequent extravasation from the leaky tumor vasculature, and the accumulation at the tumor site. Although the acquisition of images itself is non-invasive, the organs of interest first need to be exposed, and/or an imaging window needs to be implanted in the animal (Figure 10).

Tumor vascularization was first confirmed by intravenously injecting a low molecular weight dextran. Internalization of the NPs by endothelial cells results in global labeling of the entire vasculature after intravenous administration. The mean fluorescence signal in the tumor and the stroma was calculated to obtain the tumor/stroma ratio where a ratio higher than 1 indicates tumor uptake of the NPs. Visualization and quantification are possible up to 72 h with only little influence of the NPs on the host and tumor systems observed. The uptake of several NP formulations including QDs, liposomes, and iron oxide NPs was assessed using this model which highlights its potential for the in vivo analysis of the biodistribution of various NP formulations [57].

Another research study applied IVM to investigate the delivery and targeting of single-walled carbon nanotubes in a small animal tumor model. Labeling with RGD peptide ensured targeting to α_v_β_3_-integrins overexpressed on tumor vasculature and on the surface of some tumor cells. More specifically, they evaluated the circulation of NPs, their binding to tumor vasculature, extravasation, their binding to tumor cells, and tumor retention. Dorsal skinfold chambers were surgically implanted to make imaging possible. According to this study, IVM in combination with dorsal skin chambers serves as a valuable method to study vascular targeting of NPs. More insight into this mechanism would be beneficial for various therapeutic approaches, such as anti-angiogenic therapies and vascular re-normalization, both focusing on this process as a principal concept. These experiments also revealed that binding of a high number of RGD labeled NPs can reduce vessel functionality by inducing apoptosis. In addition, it was observed that circulating leukocytes represent a delivery system for these single-walled carbon nanotubes through uptake and subsequent extravasation into the tumor. This process is responsible for more than 20% of the total accumulation of NPs in the tumor at 1-day post-injection. Binding to tumor cells is a precondition for NPs to exert their effect. This study showed that up to 1 week, the control NPs showed similar binding capacity. Hereafter, both the amount of control NPs bound to tumor cells and those free in the interstitium decreased. Using IVM, Bryan Ronain Smith et al. were able to obtain data indicating that tumor cell-specific targeting ligands have a positive influence on the accumulation of single-walled carbon nanotubes in tumors only at certain time periods [58]. The same method also revealed that the binding mechanism of targeted NPs to tumor cells might be understood as an equilibrium process. Taken together, IVM can give more insight into understanding the spatiotemporal processes involved in single-walled carbon nanotubes targeting which is important to select the most optimal time points for NP-based diagnostic imaging and therapeutic treatment [58]. 

It is important to note, that for detailed, high-resolution studies at high speeds, the size of the NPs is essential in obtaining good results. To date, most studies employing IVM have used NPs with sizes of minimally 100 nm, as individual NPs as small as 10 nm would be very difficult to detect under these conditions. It is therefore important to properly set up any study using IVM to ensure that the NPs used can be readily detected and rapidly monitored. While this method showed successful results in real-time monitoring of NP biodistribution, certain research questions, such as the influence of NP size on tumor uptake, would not be easily addressed using this method.

#### 4.2.6. High-Resolution 3D Optical Mapping of Intratumoral Nanoparticle Distribution

As mentioned before, the biodistribution of NPs is still not fully understood since no suitable imaging techniques are currently available. Although prevailing imaging methods such as ultrasound, PET, and MRI can be used to acquire images of the gross distribution of nanomaterials in the body, interactions with the vasculature and other cell types cannot be visualized. In this regard, three-dimensional (3D) optical mapping offers the possibility to image the biodistribution and interactions of materials in whole organs ex vivo [92]. A major advantage of this technique is that the 3D structure of organs is preserved, in contrast to flow cytometry and immunohistochemistry, both methods currently used to study biodistribution at the cellular level. Keeping the 3D structure intact is of major importance since it includes the vasculature, lymphatic vessels, and different cell types and constitutes the primary biological barrier controlling nanomaterial transport. Three-dimensional optical mapping requires optical clearing of the tissues, using one of many available methods, and has been successfully demonstrated using CLARITY, a previously developed method by Chung et al. [108]. Primarily, the tissue is covalently bound to an acrylamide hydrogel by chemical cross-linking to ensure that nanomaterials are not lost during the clearing process. Lipids, which form a major contribution to light scattering, do not take part in the cross-linking reaction and are removed by immersing the tissue in surfactant sodium dodecyl sulfate (SDS). Electrophoresis at 25 Volt and 37 °C is performed to enhance the migration of charged SDS micelles through the tissue. Eventually, the tissue is rendered transparent, allowing light transmission. The challenge in developing this method was to define how NPs can be retained during the clearing process. Since their surface is often modified to fulfill different functions, NP formulations differ greatly from each other. Therefore, one cannot rely on these diversified functional groups to ensure retention during the purification process. A more universal approach that applies to all NP formulations, independent of their composition, is needed to avoid their removal during the clearing process. It has been shown that serum proteins adsorb onto the NP surface upon intravenous NP administration. This phenomenon is observed for all different types of NPs investigated so far. These adsorbed serum proteins are cross-linked into the tissue and enclose the NPs. Apart from separate organs, whole animals can be cleared to study the biodistribution in the entire body at the subcellular level [109]. Current commercially available clearing devices are quite expensive and clearing tissues can take up to 1 month. Both of these factors encumber the time and cost objectives of research studies and can be resolved with the 3D optical mapping technique. Sindhwani et al. successfully used 3D optical mapping to evaluate the distribution of CdSe QDs within tissues. To enable quantitative analysis, reference points such as the vasculature, nuclei, and cells were selected, and the location of the nanomaterials was represented in numbers. The spatial distribution within tissues was evaluated based on the fluorescence intensity of the nanomaterials and the number of nuclei at a given distance from the blood vessels [92].

LSM, also referred to as single plane illumination microscopy (SPIM), is another optical imaging technique that can be used to study the biodistribution of NPs, in vivo as well as ex vivo. This method allows fast and gentle 3D imaging, at single-cell resolution, of a variety of samples ranging from cells to organs and even whole organisms. LSM offers a number of advantages over other optical imaging modalities and has become a standard to study developmental biology and live organ function over time. The sample is illuminated using a laser light source placed perpendicular to the detector. A cylindrical lens expands the laser light and produces a thin light sheet illuminating a whole plane of the sample rather than a single point and thereby enabling rapid image acquisition. The illumination is restricted to a single plane of the sample at a time and each plane is illuminated only once, thus reducing photobleaching, phototoxic effects, and out-of-focus signal. These features greatly improve the signal-to-noise ratio and make LSM particularly suitable for the analysis of sensitive samples. The sensitivity of LSM mainly depends on the technique used to produce the light sheet. Sequential multiview imaging can be seen as an extension of LSM where 3D images are constructed by rotating the sample and collecting images from different angles. This approach of multi-angle imaging reduces light scattering, a common impediment of all light microscopy methods, and enables high-resolution imaging more deeply in the tissue. The sample should be immobilized during the image acquisition which can be completed through embedding in low-melting agarose. Since LSM can only be applied on transparent specimens, dense and opaque organs first need to be cleared for ex vivo analysis. The environment of the sample chamber, including temperature and CO_2_ concentration, can be controlled, enabling long-term monitoring. Up to now, many efforts have been made to improve the sensitivity and spatio-temporal resolution of LSM-based techniques by upgrading individual components or adding extra components such as a second illumination source and detector. Additionally, LSM can be easily combined with other modalities focusing on fluorescence, for instance, the coupling with fluorescence correlation microscopy to study the 3D distribution and movement of cytosolic proteins or the association with fluorescence lifetime imaging microscopy to analyze protein–protein interactions. Another remarkable advantage of LSM imaging systems is their simple design, allowing researchers to build an LSM apparatus themselves, specifically adapted to achieve personal objectives [110].

#### 4.2.7. Surface-Enhanced Raman Scattering

Another in vivo imaging system gaining more and more attention is surface-enhanced Raman scattering (SERS) [111]. As the name implies, SERS was developed based on the Raman scattering technique which relies on the inelastic scattering of photons upon excitation. An important disadvantage of Raman scattering is the very weak intensity since most of the scattered photons experience elastic scattering, also known as Rayleigh scattering, while only 1 in 10^6^ incident photons undergo inelastic or Raman scattering. As a result, useful Raman signals can be easily exceeded by Rayleigh scattering, background signal, and noise. In this regard, SERS was shown to enhance the Raman scattering from a sample and increase the sensitivity of Raman spectroscopy. The enhancement factor achieved by SERS substrates enables the acquisition of signals from samples that previously showed too weak scattering or were too low in concentration to be detectable [112]. In the last two decades, this technique has been increasingly combined with NPs as SERS substrates for medical applications of in vivo imaging. 

Although the exact mechanism of SERS is still a matter of debate, two main phenomena contribute to the enhancement, namely, electromagnetic and chemical enhancement [112]. During electromagnetic enhancement, plasmon oscillations perpendicular to the metal surface results in two consecutive enhancements: first, of the excitation light, and second of the Raman scattered light. Moreover, the localized surface plasmons, occurring at so-called “hotspots”, can be further optimized depending on the SERS substrate design. Chemical enhancement, on the other hand, suggests inter- and intra-molecular charge transfers leading to the greatest enhancement for molecules adsorbed to the metal surface.

Most conventional SERS-active materials are metallic NPs including gold (Au), silver (Ag), or copper (Cu) [111]. The popularity of these substrates can be attributed to their low cost, simple preparation, and surface plasmon resonance in the NIR region, where most Raman detection occurs. Furthermore, QDs harbor some additional benefits compared to traditional SERS-active NPs such as their size and special optical properties. Synergistic SERS effects can be achieved by using hybrid structures, such as gold–silver nanoshells and graphene oxide-wrapped gold nanorods.

As an example, Zavaleta et al. injected five different SERS NPs intravenously into a living mouse to image their natural accumulation in the liver [113]. The SERS NPs consisted of an Au core with a diameter of 60 nm, coated with a monolayer of a Raman-active organic molecule, and encapsulated with a 30 nm diameter silica shell resulting in a particle of around 120 nm. Due to this size range, the particles tend to get taken up by Kupffer cells of the reticuloendothelial system and, as a result, naturally accumulate in the liver. They imaged the mice at different time points post-injection 1, 24, and 48 h. Results show successful identification and spectral separation of all five types underlining the great potential of SERS for the deep tissue in vivo multiplexed imaging to simultaneously detect multiple biomarkers associated with a specific disease.

#### 4.2.8. Background-Free Photoacoustic Imaging

Another relatively new imaging technology that allows monitoring in vivo NP biodistribution is photoacoustic imaging (PA). It provides images in three dimensions with high spatial resolution. The principle of this technique relies on the photoacoustic effect. In short, photons are reflected by tissue, then transduced and registered as ultrasonic waves, and finally converted to images. When compared with regular optical imaging, PA imaging offers the advantage of less scattered ultrasonic waves, compared to photons, and will therefore result in higher spatial resolution and improved imaging depth. Compared to pure ultrasound imaging, PA imaging delivers improved tissue contrasts. There are two modes of PA: either purely relying on the endogenous tissue contrast, or injecting (exogenous) imaging contrast agents [114,115].

Despite the approval of only some (small molecular) imaging dyes for human use, multiple other contrast agents, mostly (gold) NPs, are of interest for PA imaging [114]. A common drawback of combining PA imaging with any type of contrast agent is the interference of signals from the contrast agent with endogenous background signals originating from optical absorbance by biomolecules (e.g., hemoglobin, melanin, etc.) [116].

In a study from 2019, researchers have come forward with a system where Au NPs are packaged into microbubbles, which upon ultrasound delivery, will result in bursting the microbubbles and allowing the Au NPs to form aggregates with the remainder of the microbubbles, delivering an enhanced near-infrared PA signal. The background PA signals could simply be removed by subtracting the PA image captured a post-ultrasound-induced burst of the microbubbles from the PA image captured before the burst. This ultimately enables one to register a highly sensitive background-free photoacoustic image of the location of the Au NPs [116]. 

In a clinical setting, PA imaging can be combined with the therapeutic characteristics of the applied contrast agents (theranostic). For example, gold nanoparticle-mediated hyperthermia has been shown to have great potential in animal studies and early phase clinical tests. Here, PA imaging can be of use to monitor the treatment in real-time and with high spatial contrast [117].

Furthermore, PA imaging can be applied in a dual-mode setting. Combined with fluorescence it was used to monitor nanocomposites as a theragnostic agent in an MDA-MB-231 tumor mouse model. After injecting the nanocomposites into the tumor, the target area was precisely diagnosed by fluorescence/photoacoustic guidance, followed by a 20-day NIR laser irradiation treatment, resulting in a tumor disappearance [118].

### 4.3. Monitoring Therapeutic Efficacy and Delivery

Optical imaging offers the ability to monitor the efficacy of therapeutic agents and their specific delivery at the preclinical level. However, to make this possible, the cells involved in the pathogenesis of interest first need to be genetically modified to obtain luminescent or fluorescent properties. One method to achieve this is through transduction with vectors containing at least a constitutive or inducible promoter and a reporter protein. Extra DNA sequences of proteins expressing other functionalities, such as selection markers, can also be added to the vector. Apart from the transduction of cells, the development of a transgenic animal model also results in cells with luminescent or fluorescent properties. In this case, the transgene can be expressed in the whole animal, or the expression can be limited to a specific type of tissue. Hereafter, the influence of therapy can be evaluated by measuring the luminescence or fluorescence signal intensities, representing reporter gene expression in the cells of interest. From a practical point of view, to evaluate the therapeutic efficacy of, for example, an anticancer agent, the total flux per cell in vivo, expressed as photons/second, should first be determined by injecting a known number of transduced tumor cells in the animal and measuring the luminescence output [8]. Hereafter, the effect of the anticancer drug on the tumor can be monitored by measuring the luminescence intensity after treatment where higher and lower BLI signals, respectively, correlate to an increase and a decrease in tumor volume [8]. The same methodology can also be used to study other pathological conditions such as, for example, hypoxia and inflammation. To detect hypoxia, the vector used for transduction should contain an inducible promotor specific for hypoxia, such as HIF1α, to ensure that the reporter protein is only expressed in cells experiencing hypoxia. This methodology offers to develop an optical imaging method allowing us non-invasively and with high sensitivity to monitor the efficacy of therapeutics in a high throughput manner. In addition to evaluating treatment efficacy, the specific delivery of therapeutic agents to certain tissues or cells can also be monitored using optical imaging. In this case, both the agent and the targeted cells should be labeled with a luminescent or fluorescent probe.

As mentioned in the introduction, some studies focus on the toxicity of NPs themselves to attack cancer cells instead of using them as carriers for other therapeutic compounds [8]. The delivery, and in some cases also the therapeutic efficacy, of NPs, used as a therapeutic agent and showing luminescence properties on their own or after modification can also be monitored in vivo by measuring luminescence output. Recent studies investigate the capacity of CdTe QDs to kill cancer cells through the “lysosome-enhanced Trojan horse effect” [8]. QDs are very small semiconductor particles with fluorescent properties making them suitable for optical imaging. The release of Cd^2+^ ions and consequently their toxicity can be assessed by measuring the decrease in fluorescence signal, stemming from the excitation of the QDs. Data revealed that this decrease correlates with therapeutic efficacy. Although these types of QDs showed evidence for the “lysosome-enhanced Trojan horse effect” in vivo, they are also associated with toxic effects on liver and kidney function when administered intravenously. Even other administration routes cannot assure their safety for longer treatments. Therefore, NPs consisting of other ions should be tested as well. Silver NPs, for example, are currently tested in clinical studies for their ability to kill cancer cells [8].

#### 4.3.1. Image-Guided Surgery

Despite several advances in cancer therapies, surgical resection remains one of the most frequently used treatment approaches for solid tumors. However, long-term survival rates after surgery are still disappointing with reported tumor recurrence of 20–30% as well as the development of postoperative metastasis. While precise and complete removal of the tumor, including the primary tumor at the macroscopic level and potential microscopic residual lesions in neighboring tissue and draining lymph nodes, greatly determines the clinical outcome, surgeons rely solely on their subjective observation using visual inspection and palpation for intraoperative evaluation. Optical probes for image-guided surgery (IGS) face multiple challenges that need to be addressed including deep tissue penetration and high brightness, high SBR ratio, sufficient surgical time window, and rapid clearance from the body. While adequate tumor accumulation is essential to provide an optimal time window, this often results in a trade-off between a long tumor retention time and rapid clearance [119,120]. Here, nanotechnology can play an important role in facilitating IGS by offering minimally invasive visualization and surgical navigation and thereby enhancing preoperative tumor detection, tumor margin delineation as well as intraoperative minimal residual diseases discovery, and postoperative complete resection verification. As cancer metastasis often occurs through the lymphatic system, sentinel lymph node (SLN) mapping is a crucial part of tumor surgery. The gold standard for SLN visualization, using a radionuclide combined with a dye, is not as common in the clinic as a result of undesirable radiation exposure. Various NMs have been investigated to offer a radioisotope-free approach for SLN detection for example, superparamagnetic iron oxide nanoparticles (SPIONs), which already showed an improved accurate diagnosis of LN metastases, preoperatively and intraoperatively [119].

As discussed previously, imaging using the NIR window reduces tissue scattering, absorption, and autofluorescence compared with the visible spectrum allowing deeper tissue penetration. Therefore, NMs exhibiting NIR emission can be used to provide guidance for surgical resection of small tumors in the early stages and thereby improve the post-treatment survival [98]. Given that indocyanine green (ICG) is the only FDA-approved NIR fluorescence agent, researchers have been focusing on the development of NIR NP-based fluorescent probes with increased tissue penetration for application in different fields including ICG [119]. In this context, RE NPs, composed of 15 lanthanides, were previously reported as promising NIR fluorophores showing exceptional advantages including high NIR luminescence efficiency, low toxicity, good biocompatibility, high chemical/photochemical stability, tunable emission, large Stokes shift, and sharp emission peaks. In addition, various approaches can be applied to endow them with NIR-emission, degradability in physiological fluids, and even long lifetime “after-glow” persistent luminescence. For example, Zhang et al. described the in vivo assembly of NIR-II emissive RE NPs which were successfully used for the complete removal of metastases smaller than 1 mm in IGS at the preclinical level [120]. Although RE NPs clearly show promising potential in a wide range of applications, NIR small molecular dyes, and QDs still remain the most commonly used NIR fluorescence probes for IGS. Two important reasons contributing to the low usage include safety issues related to the release of RE ions and the fact that smaller RE NPs are needed to achieve higher sensitivity and resolution but, by reducing their size, the luminescence efficiency inherently decreases. Additionally, studies using lanthanide NPs are often conducted in small and simple animal models such as mice while these formulations have not yet been tested in larger animals with thicker tissues that more closely mimic the clinical practice in humans. Given that the currently reported penetration depth of NIR fluorescence does not usually exceed 10 mm, clearly hampering its use in humans, further improvement of NIR-emissive RE NPs is urgently needed [120].

While NMs can undoubtedly contribute to precise surgical resection by delineating tumor margins, integrating different imaging modalities in a multimodel imaging platform and the importance of pre- or postoperative, neoadjuvant, or adjuvant strategies including but not limited to chemotherapy, radiotherapy, immunotherapy, PDT and PTT, for synergistic cancer therapy should be emphasized [119].

#### 4.3.2. Theranostics Development

**Figure 11:**
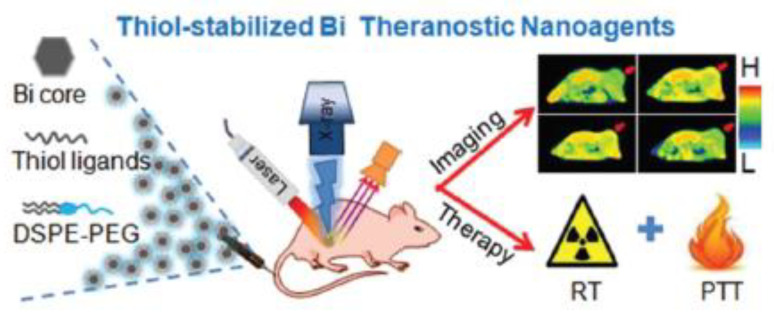
A theranostic nanoplatform synthesized from thiol-capped Bi nanoparticles. A high X-ray attenuation coefficient and a strong photothermal conversion efficiency endow these NPs with simultaneous CT imaging, radiotherapy, and thermotherapy. This image has been reproduced with permission from Yu et al. [121]. © 2018, Elsevier.

Another promising strategy, mainly focusing on cancer research, is the development of NPs with diagnostic potential as well as therapeutic efficacy, also referred to as theranostics (Figure 11).

**Figure 12:**
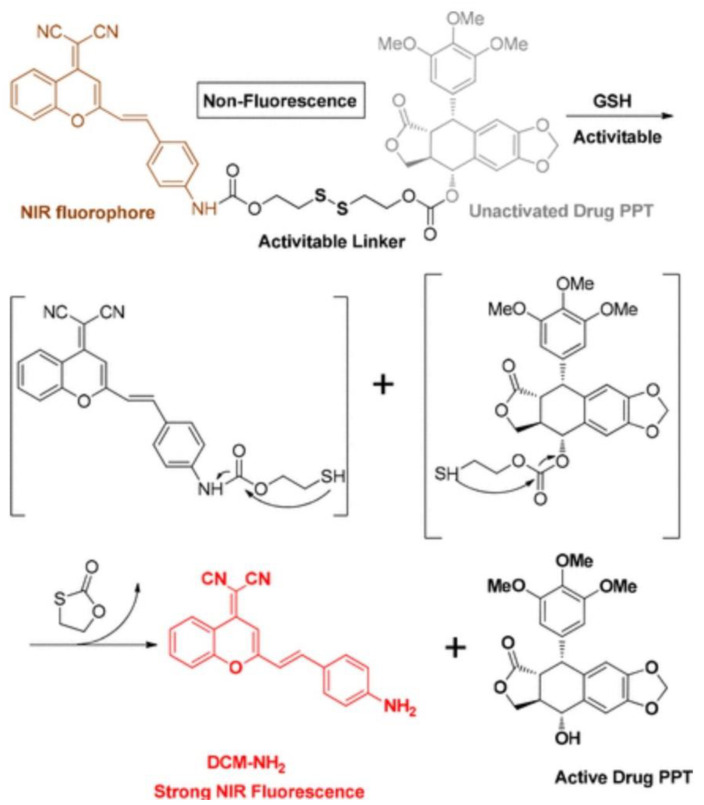
Proposed GSH-induced activation mechanism of the theranostic NIR fluorescent anticancer prodrug system. Cleavage of the disulfide bond by thiols such as GSH induces release and activation of the anticancer drug and simultaneously enhances NIR fluorescence. This image has been reproduced with permission from Lui et al. [122] © 2017, American Chemical Society.

The combination of such two functional properties in a single nanostructure allows accurate tumor imaging by tracking the accumulation of therapeutic agents within the tumor as well as subsequent eradication [78]. Many currently applied therapeutic approaches such as chemotherapy, immunotherapy, and PTT have been investigated for their opportunity to enter the field of theranostics. However, until now not a single NP has been approved by the FDA for theranostic applications [6,78]. One of the main reasons for this little clinical interest and use is probably due to the fact that for most of these formulations, the properties of an imaging agent and those of a therapeutic agent do not always match. For example, the biodistribution and kinetics for both types of agents are typically not the same to reach either optimal imaging contrast or therapeutic efficacy. For this reason, the synthetic procedure is highly complex, and it is often a compromise to make a theranostic agent rather than making a more powerful tool. One strategy to avoid this drawback is the development of “all-in-one” nanoplatforms where imaging and therapeutic properties are merged into a single agent. Naphtalocyanine-loaded poly(ethylene glycol)-block-poly(ε-caprolactone)-based NPs are a promising example of such a formulation where absorbed energy is effectively converted into fluorescence, heat, and ROS enabling image-guided combinatorial phototherapy [78]. Another design approach that is widely investigated to obtain NPs with theranostic potential and which also circumvents the above-mentioned trade-off is the development of activatable fluorescent prodrug systems. In recent years, theranostic prodrugs have gained attention as a promising platform for the real-time in vivo monitoring of the biodistribution and activation of prodrugs. In general, this platform consists of a compound of interest covalently coupled to a fluorescent reporter through a cleavable linker **(**Figure 12). Cleavage of the linker results in concomitantly drug release and fluorescence generation.

Prodrugs are particularly interesting since they minimize unwanted side effects, allow controlled drug release and improve therapeutic efficacy. Currently, dicyanomethylene-4H-pyran (DCM) is often used as a fluorescent reporter as its emission spectrum is in the NIR region allowing deeper penetration depth and reducing background interference. In addition, DCM exhibits high photostability which is an important feature for theranostic prodrug systems. The selectivity and efficacy of cancer therapy can be further improved by choosing a linker with a chemical structure that is activated specifically in the tumor microenvironment. Disulfide bonds are often used as a linker and can be specifically cleaved by reducing thiol-containing structures such as glutathione (GSH) in a dose-dependent manner. GSH functions as an important antioxidant in organisms and is abundantly present in hypoxic environments, typically associated with tumors. The intracellular concentration of GSH is considerably higher in tumor cells compared to normal cells and the concentration in the plasma is very low assuring specific cleavage in tumors. Three examples of theranostic drug delivery systems that are already proven successful for in vivo imaging and therapy are composed of podophyllotoxin or camptothecin as anticancer drugs, DCM as NIR fluorescent reporter, and 2,4-dinitrobenzenesulfonyl or a disulfide bond as thiol responsive group [122,123,124]. Encapsulation of these constructs in PEG-coated NPs was essential to acquire aqueous solubility and enhance the biocompatibility of the prodrug. Modification with PEG also resulted in a slower blood clearance, an enhanced passive targeted delivery to the tumor through the EPR effect, and a higher antitumor activity. Upon injection, the NPs are taken up by the endocytosis pathway whereafter the active drug is released in the cytoplasm via intracellular cleavage. No interference of other biologically relevant molecules including amino acids and metal ions was observed in either of these three theranostic prodrug systems, showing that the disulfide bond cleavage and subsequent drug release is selective for thiol-containing species. Additionally, cleavage only occurs in thiol-containing tissues, which also reduces side effects. These thiol-responsive theranostic prodrug systems provide a valuable strategy to further optimize and expand theranostic drug delivery systems and to enable simultaneous tumor imaging and therapy [122,123,124].

## 5. Conclusions

Although nanomedicine is a relatively new discipline, the field is rapidly expanding and promising designs are quickly entering preclinical and clinical trials [7]. As discussed above, NPs really have the potential to fully revolutionize the health care approach mainly explained by the opportunity to integrate multiple functions in a single nanostructure making them suitable for a wide range of applications [125]. To further boost the translation of NMs to the clinical setting, all physiochemical properties related to the pharmacokinetics and dynamics of NPs, including the toxicity profile, distribution, metabolism, and excretion, should be clearly understood. So far, only 50 NMs have been approved by the FDA regardless of their strong representation in preclinical and clinical trials. This remarkably low number can partly be explained by the lack of adequate research methods to broadly investigate the biodistribution and safety profile of the newly designed NP systems [6]. The development of multifunctional NPs is also slowed down by the need for specialized equipment and advanced chemistry, as their production is often associated with technical difficulties, challenging upscaling processes, and high costs [8]. This review highlights various optical imaging techniques that provide an excellent platform to study the biodistribution of highly modifiable NPs and which can further promote the clinical translation of NMs in different applications.

The use of optical imaging tools offers diverse advantages to study the biodistribution of NPs; however, some remarks should be taken into account. A first limitation is associated with IVM where NPs cannot be too small. If they are really nanoscale (e.g., 5 nm), they may simply be too small to be detected by such systems which are mainly suited for particles that are at least 100 nm in size. Light scattering is another important limiting factor for IVM, and one should carefully consider which imaging modality is most appropriate depending on the depth of the structures of interest below the surface. In addition, efforts should be made to stabilize the organs of interest since the heartbeat, respiration, and peristaltic behavior can cause motion artifacts [126]. Another remark that should be emphasized is related to the ex vivo analysis of whole organs and concerns the selection of the correct optical clearing method rendering the tissue transparent while not affecting the NPs. This choice often depends on the NP design and modifications where additional compounds such as fluorophores and lipids can be added to the NP surface or embedded in the core. In this regard, it should be noted that for different types of NPs, different clearing methods may be more suited.

## 6. Further Improvements and Future Perspectives

Despite the great potential of the research methodologies discussed above, several limitations are faced, of which a large part will most likely be resolved by the continuous efforts that are made in the optimization of nanomaterial compositions. In this section, we would therefore like to highlight some of the most important challenges that remain to be tackled in order to benefit from the full potential of the (combined) application of nanoformulations and imaging technologies and as such, improve the further translation towards their use in the clinic.

The first challenge that has to be resolved to broaden the applicability of NPs is their targeting capacity. Most current studies achieve delivery of around 1% to the region of interest. Often, the main focus lies on the characteristics of the NP itself, such as the size and the shape, and despite interesting progress in the field [127]. However, in the majority of studies, too little attention is paid to biological parameters. For example, as it is known that the level of angiogenesis differs among tumor types, so will the accessibility for NPs. To improve the delivery of NPs to the region of interest, one should consider the nature of the tumor and its size. Since NPs are often associated with high levels of toxicity, ameliorating the specific delivery of NPs to the region of interest would make intravenous administration a more rational option [8]. Another way to circumvent their high toxicity toward healthy tissue and thereby improve the use of NMs as anticancer therapy is to design NPs that only exert toxic effects under conditions specifically associated with tumor cells. As such, it is known that the pH in the tumor microenvironment is lower than in healthy tissue and this fact can be exploited to design NPs with specific pH-dependent dissolution kinetics which release toxic ions, particularly near tumor tissue.

A second challenge to overcome, apart from the targeting capacity of NPs, is their blood circulation lifetime. One of the main factors that influence the in vivo effectivity of NPs concerns the pharmacokinetics rather than the internal structure. Most NPs are rapidly cleared from the bloodstream upon recognition by macrophages from the MPS. Although various approaches have already been investigated to increase their blood circulation lifetimes, such as PEG coating, shape modification, or size reduction, these adaptations imply important restrictions for the engineering part and detract from their multifunctional character. Another alternative explored to avoid the rapid removal from the bloodstream is cellular hitchhiking, in which various cell types serve as carriers for NPs. Recent studies involving RBC-hitchhiking, suggest the effectiveness of NP delivery does not critically depend on the circulation lifetime as a high degree of NP accumulation in the target tissue could be observed after only a few minutes. These results are in strong contrast with the existing perception that their rapid removal from the blood circulation subordinates them to molecular substances [128].

Other future efforts to be made that could greatly expand the applicability of NPs in the clinic and which should be exploited more in the future include the development of organic phosphorescent NPs, NP-based contrast agents, theranostics, and the combination of NP-based therapeutics with immune therapy. As explained before, ultralong phosphorescence or afterglow imaging provides a valuable and highly sensitive tool for longitudinal imaging and lymph node mapping in vivo. Unfortunately, afterglow is known to be a characteristic of mainly inorganic NPs, which often conflicts with the safety of these NPs. Therefore, Xu Zhen et al. developed a water-soluble organic semiconducting NPs [94]. The same top-down approach was used to enhance the phosphorescence of different semiconducting dyes emphasizing the flexibility and generalizability of this method. Since, in this approach, the formation of H-aggregates, where molecules are stacked predominantly face-to-face [129], is considered a key point characteristic allowing prolonged phosphorescence, researchers should focus on other molecules, likely to form aggregates, to develop novel optical probes for in vivo imaging. In addition, alternative synthesis methods further enhancing the formation of H-aggregates should be explored to enable additional prolongation of the phosphorescence [94].

Another widely explored topic related to the combination of NPs and imaging techniques is the development of NP-based imaging probes and contrast agents, primarily important to guide diagnostic tumor imaging, define optimal time points for therapeutic treatment, and to evaluate the specific delivery [58]. For example, NPs with optical imaging properties can help surgeons in accurately delineating tumor margins and identifying residual lesions intraoperatively, offering great potential to improve patient care since surgery still remains the most effective approach to treating cancer [125]. Furthermore, it has been illustrated that NP-based contrast agents offer an enhanced signal intensity, targeted delivery, and blood circulation lifetime compared to traditional contrast agents. These improved parameters are mainly observed in the field of cancer diagnosis. Despite large efforts in these fields over the past years, only a few NP-based contrast agents have been assessed in clinical trials [6].

We would also like to highlight the development of theranostic formulations. As mentioned above, theranostics, especially for cancer, have a great potential to improve therapeutic efficacy by realizing personalized medicine via image guidance. However, the clinical translation of such systems is partly slowed down by the complexity of the design and the inevitable trade-off that needs to be made between the optimal parameters of both agents. Here, structures possessing both imaging as well as therapeutic properties, pro-drugs particularly, are good candidates to focus on as they eliminate the need to compromise [125].

To conclude, a multidisciplinary approach driven by the combined knowledge and advances in the fields of chemistry, pharmacy, biology, medicine, physics, and engineering will be of great benefit for the combined application of nanomaterials with (optical) imaging technologies and the expansion of NP use in various other biomedical applications [6].

## Data Availability

Not applicable.

## List of Abbreviations

3D	Three-dimensional
BASFI	Bioluminescence assisted switching and fluorescence imaging
BioLeT	Bioluminescent enzyme-induced electron transfer
BLI	Bioluminescence imaging
BRET	Bioluminescence resonance energy transfer
CCNEs	Centers of Cancer Nanotechnology Excellence
CNTs	Carbon nanotubes
CT	Computed tomography
CTLs	Cytotoxic T lymphocytes
DCM	Dicyanomethylene-4H-pyran
DCNPs	Down-conversion nanoparticles
E.C.	European Commission
EGFR	Epidermal growth factor receptor
EMA	European Medicines Agency
EPR	Enhanced permeation and retention
ErNPs	Erbium-based rare earth nanoparticles
ETPN	European Technology Platform on Nanomedicine
EUNCL	European Nanomedicine Characterization Laboratory
FA	Folic acid
FDA	Food and Drug Administration
FLECT	Fluorescence emission computed tomography
FR	Folate receptors
FUEL	Fluorescence by unbound excitation from luminescence
GBM	Glioblastoma multiforme
GMP	Good Manufacturing Practice
GNP	Gold nanoparticles
GSH	Glutathione
HiPCO	High-pressure carbon monoxide conversion
ICG	Indocyanine green
IGS	Image-guided surgery
IL-2	Interleukin-2
IVM	Intravital microscopy
LETH	Lysosome-enhanced Trojan horse
LSM	Light sheet microscopy
LV	Laser vaporization
MPI	Magnetic particle imaging
MPS	Mononuclear phagocyte system
MRI	Magnetic resonance imaging
MWCNTs	Multi-walled carbon nanotubes
NCI	National Cancer Institute
NCL	Nanotechnology Characterization Laboratory
NIR	Near-infrared
NIST	National Institute of Standard and Technology
NMs	Nanomedicines
NP	Nanoparticle
NPs	Nanoparticles
PCA	Principal component analysis
PD-L1	Programmed death-ligand 1
Pdots	Polymer dots
PDT	Photodynamic therapy
PEG	Polyethylene glycol
PET	Positron emission tomography
PLGA	Polylactide-co-glycolic acid
PPV	Poly(p-phenylenevinylene)
PTT	Photothermal therapy
RE	Rare earth
ROS	Reactive oxygen species
SBR	Signal-to-background ratio
SDS	Sodium dodecyl sulfate
SLN	Sentinel lymph node
SPIM	Single plane illumination microscopy
SPIONs	Superparamagnetic iron oxide nanoparticles
SPNs	Semiconducting polymers nanoparticles
SWCNTs	Single-walled carbon nanotubes
TAB	Translation Advisory Board
T/NT	Tumor-to-normal tissue
QDs	Quantum dots
